# Heterologous expression of a cold-active small esterolytic enzyme from *Pseudomonas sivasensis* R11S16 and its potential for azithromycin removal

**DOI:** 10.1007/s13205-026-04884-y

**Published:** 2026-06-01

**Authors:** Gizem Çon, Emre Karakaya, Hayrettin Saygin, Salih Saricaoğlu, Serpil Könen Adıgüzel, Ali Osman Adıgüzel

**Affiliations:** 1 Department of Molecular Biology and Genetics, Ondokuz Mayıs University, Samsun, Turkey; 2 Department of Biology, Ondokuz Mayıs University, Samsun, Turkey; 3 Stem Cell Research and Application Center, Ondokuz Mayıs University, Samsun, Turkey

**Keywords:** Esterase, *Pseudomonas sivasensis*, Cold-active, Azithromycin removal

## Abstract

The gene encoding a hypothetical YqiA/YcfP family α/β-fold hydrolase from *Pseudomonas sivasensis* R11S16 was cloned, heterologously expressed in *Escherichia coli*, and purified as a histidine-tagged recombinant enzyme (PsEst_yfh_). The enzyme, with a molecular weight of about 23 kDa, exhibited typical esterase activity against short-chain *p*-nitrophenyl esters (C_4_ > C_8_ > C_2_ > C_10_). PsEst_yfh_ exhibited the highest activity at 20 °C and pH 9, indicating its cold-active and alkaline-tolerant nature. The half-life of PsEst_yfh_ at 30 °C and 40 °C was nearly 6 h and 2 h, respectively. The enzyme retained about 70–95% of its activity after 1 h of incubation over the pH range of 5–10 and remained remarkably stable, with ~ 50% relative activity even in the presence of 16% NaCl. In the presence of 1 mM Ni²⁺, Cu²⁺, and Ca²⁺, the enzyme activity increased by 26.74%, 24.20%, and 20.84%, respectively. The enzyme retained more than 80% of its activity in the presence of 10% (v/v) methanol, acetone, chloroform, and butanol. The Michaelis constant (*K*_*m*_) and the maximum velocity (*V*_*max*_) of PsEst_yfh_ were determined from a Lineweaver-Burk plot to be 129.97 µM and 33.3 µmol/min, respectively, with *p*-NPB as the substrate. Importantly, PsEstyfh achieved over 95% reduction in azithromycin’s antimicrobial activity. Furthermore, ecotoxicological assessment indicated that the resulting transformation products did not exert significant toxic effects on *Lemna minor*. To our knowledge, this study represents the first report describing the recombinant production, functional characterization, and biotechnological potential of an esterolytic enzyme encoded by a hypothetical YqiA/YcfP family α/β-fold hydrolase gene from *P. sivasensis*.

**Supplementary Information:**

The online version contains supplementary material available at 10.1007/s13205-026-04884-y.

## Introduction

The uncontrolled and improper use of antibiotics for human and animal treatment, as well as for food preservation purposes, has led to a rapid increase in antimicrobial resistance (AMR) on a global scale and has become a critical threat to public health (Patra et al. 2025). Antibiotic resistance not only limits treatment options for patients but also imposes a serious economic burden on healthcare systems by increasing morbidity, mortality, and hospital stays (Mohan et al. 2025). It is estimated that resistant infections cause more than 35,000 deaths annually in the US alone, and if current trends continue, global annual deaths due to antibiotic resistance could exceed 10 million by 2050 (Eke and Cua 2025). Furthermore, the global economic loss due to AMR is reported to exceed $100 billion (Bapat et al. 2022). Antibiotics released into the natural environment accelerate the development and spread of resistance genes by creating selective pressure even at low concentrations, thus establishing a direct relationship between environmental antibiotic pollution and antibiotic resistance. In this contexts, despite the implementation of national action plans to limit antibiotic use in many countries, population growth, continued antibiotic use in veterinary and industrial animal husbandry, and the resulting spread of antibiotic residues into the environment are increasing the frequency of resistant microorganisms in populations (Browne et al. 2021; Cedeño-Muñoz et al. 2024). Therefore, the removal of antibiotics from wastewater has become a highly important issue.

Conventional wastewater treatment plants (WWTPs) primarily focus on removing organic matter and nutrients, such as nitrogen and phosphorus, and are often inadequate for removing micropollutants, including antibiotics (Oharisi et al. 2023; Addis et al. 2024; Wang et al. 2025a). Low-molecular-weight antibiotics, in particular, are poorly removed in WWTPs, leading to antibiotic accumulation in receiving water environments and the selection of resistant microorganisms (Ezra et al. 2025). To date, it has been widely reported that antibiotics have been detected in WWTP effluents at levels of ng/L to µg/L, and that this burden poses serious ecotoxicological risks to river and lake ecosystems (Singh et al. 2025). Although advanced oxidation processes, electrocoagulation/flocculation, membrane filtration, and adsorption are suggested as additional treatment technologies to address this issue, high costs, energy requirements, by-product formation, scalability, and sustainability issues limit their widespread applicability (Saini et al. 2026). Therefore, in recent years, enzymatic treatment approaches have emerged as a promising alternative for antibiotic removal due to their low energy requirements, environmentally friendly nature, and high catalytic activity (de Boer et al. 2025; Gao et al. 2025).

Esterases (EC 3.1.1.1.x) are a group of enzymes with high potential for the removal of macrolide antibiotics (Dhindwal et al. 2023). Esterases are enzymes that mainly convert esters into carboxylic acids and alcohols. They also act on esters in thioesters, phosphoesters, amides, and epoxides (Rafeeq et al. 2022). They basically catalyze the breaking and making of carboxyl ester bonds. Although they are quite similar to lipases in terms of structural and functional properties, they differ from lipases in that their specificity for short-chain esters (< C8) is higher than that of long-chain esters (> C8) and they do not require interface activation, i.e., they can hydrolyze water-soluble esters (Mussakhmetov and Silayev 2025). Their active sites do not form a hydrophobic area as in lipases. Alternatively, they have an acyl-binding site in their active site to bind the substrate (Noby et al. 2022). Unlike lipases, esterases do not require a cofactor for their activity (Mussakhmetov and Silayev 2025). Structurally, these enzymes with an α/β folding motif are classified as the serine hydrolases and contain the catalytic triad serine (Ser), aspartic acid (Asp), and histidine (His) in their active sites (Barzkar et al. 2021). Glycine (Gly) and other small molecules in the region where the oxyanion hole is formed around the serine in the active site are conserved to keep the enzyme-substrate association stable. Typically, these enzymes have the consensus sequence Gly-X-Ser-X-Gly (Gly: glycine, X: any amino acid), in which the serine in the active site is located (Rafeeq et al. 2022). In the first step of esterolytic catalysis, serine in the active site undergoes a nucleophilic attack on the carbon atom in the carbonyl functional group of the substrate to form a tetrahedral intermediate stabilized by His and Asp. Then, due to the proton transfer between His and Asp, a rearrangement occurs in the whole molecule and an alcohol is released from the tetrahedral intermediate. In parallel, a covalent bond occurs between the acyl substrate and the enzyme, and an acyl-enzyme complex is formed. The hydrolyzed water (activated by His) acts by nucleophilic attack on the acyl-enzyme complex, forming a second tetrahedral intermediate (carboxylate). Finally, histidine provides a proton to the oxygen atom of Ser, and the covalent bond between Ser and the acyl compound is broken. Thus, a carboxylic acid is released while the enzyme is restored (Aranda et al. 2014).

In addition to esterase enzymes, it is known that different enzyme classes, such as laccase, peroxidase, and β-lactamase, can also be used in antibiotic degradation; however, the individual use of these enzymes shows limited effectiveness against the wide variety of antibiotics found in real wastewater environments due to their high substrate specificity (Nawaz et al. 2024; Adıgüzel et al. 2025; Jiang et al. 2025). Thus, fusion enzyme approaches, which combine multiple catalytic functions in a single biocatalytic system, emerge as a more viable strategy due to their potential to simultaneously affect different classes of antibiotics. In designing fusion enzymes, the relatively small molecular size of the catalytic components facilitates the genetic construction process and increases the probability of proper folding and functional expression (Elleuche 2015). Therefore, esterases with esterolytic activity and relatively small structures are particularly advantageous as core components in fusion enzyme architectures. In this study, a hypothetical “YqiA/YcfP family alpha/beta fold hydrolase” was evaluated as a suitable candidate for fusion enzyme design to expand the catalytic scope for macrolide antibiotics due to its small molecular weight, bioinformatics-based distinct esterolytic potential, and structure composed of a small number of amino acids.

There are many esterases that are effective in removing antibiotics. However, most of them do not possess the biochemical properties suitable for real wastewater treatment applications. The main reason for this lies in the wastewater’s physicochemical properties. The alkaline discharge water from WWTPs is usually cooled before being released into aquatic ecosystems. Therefore, it is desirable that enzymes capable of removing antibiotics from WWTPs discharge water be effective at relatively cold temperatures of 15–20 °C and at a pH range of 8–9 (Martin and Vanrolleghem 2014). However, it has been observed that many esterase enzymes reported to have antibiotic removal potential do not possess these characteristics. For example, *Pseudomonas* sp. GD100 esterase showed good activity under neutral and alkaline conditions, but it was determined that the maximum activity temperature was 50 °C and that there was a significant decrease in activity parallel to the decrease in temperature (Kim et al. 2002). Therefore, a psychrotolerant *Pseudomonas* isolate was selected as the source of the YqiA/YcfP family alpha/beta-fold hydrolase gene.

In summary, within the scope of this study, a psychrotolerant *Pseudomonas* sp. R11S16 isolated from the Kaçkar Mountains was identified based on its 16 S rRNA gene sequence, and its YqiA/YcfP family alpha/beta fold hydrolase gene was cloned into a suitable expression vector and heterologously expressed in *Escherichia coli*. The biochemical and catalytic properties of the recombinant enzyme were characterized in detail, and its potential for use in antibiotic degradation was evaluated. In this regard, the presented study constitutes a first in the literature, as it comprehensively reveals the functional properties of a hypothetical YqiA/YcfP family α/β fold hydrolase enzyme and evaluates its potential for use in the biocatalytic degradation of macrolide antibiotics.

## Materials and methods

### Materials and reagents

Enzymes, buffers, and nuclease-free water used in cloning were purchased from Thermo Fisher Scientific (Waltham, MA). Glycerin, bovine serum albumin (BSA), Coomassie Brillant Blue R-250, β-merkaptoetanol (βME), sodium dodecyl sulfate (SDS), acetic acid, orthophosphoric acid, solvents, and *p*-nitrophenol (*p*-NP) linked substrates were obtained from Sigma-Aldrich (Darmstadt, Germany). Medium and buffer components were procured from Merck (Darmstadt, Germany). Ethylenediaminetetraacetic acid (EDTA) was purchased from PanReac Applichem (Barcelona, Spain). Other reagents and laboratory equipment (glass/plastics) were obtained from Tekkim (Bursa, Turkey), Biobasic (Toronto, Canada), AnalytiChem (Zedelgem, Belgium), and Condalab (Madrid, Spain) unless otherwise stated.

### Molecular identification of bacterial strain

A psychrotolerant strain, designated as R11S16, was isolated from a water sample collected from the Kaçkar Mountains (GPS coordinates, 40°52′ 54.01″ N, 41°08′ 23.26″ E). R2A agar was used as isolation media, and isolate R11S16 was purified by repeated streaking on Nutrient agar (Karakaya 2025). Genomic DNA was extracted from an overnight culture using PureLink^®^ DNA Isolation Kit (Invitrogen, USA) according to the manufacturer’s instructions. The 16 S rRNA gene was amplified by PCR using universal bacterial primers (27 F and 1525R). The PCR products were purified and sequenced using an ABI PRISM 3730 XL automatic sequencer with the forward primer 518 F (28) (Buchholz-Cleven et al. 1997) and the reverse primer 800R (Chun and Goodfellow 1995).

Chromatogram files in ABI format were checked using Chromas version 1.45, and the primers overlapped to obtain the 16 S rRNA gene nucleotide sequence in FASTA format for isolate R11S16. The resultant 16 S rRNA gene sequence was deposited in GenBank under accession number PP213417. The 16 S rRNA gene sequence was uploaded onto the EzBioCloud server (Chalita et al. 2024) and pairwise sequence similarities of the nearest phylogenetic neighbours determined. Phylogenetic trees were generated using the MEGA X software package (Kumar et al. 2018) using the neighbor-joining, maximum-parsimony (MP), and maximum-likelihood (ML) methods. The topologies of the resultant trees were evaluated by bootstrap resampling with 1000 replicates after Felsenstein (Felsenstein 1985). Since the primary focus of this study was functional characterization of a cold-active esterase, genome-level taxonomic analyses were not pursued.

### Cloning of the YqiA/YcfP family alpha/beta fold hydrolase gene

Oligonucleotide primers with an XhoI cut site (italic) at both ends were designed based on the *Pseudomonas sivasensis* type strain genome data, with an XhoI cut site (italic) at both ends for the YqiA/YcfP family alpha/beta fold hydrolase gene. Restriction enzyme recognition sites of the gene were investigated using NEB cutter v3.0 (https://nc2.neb.com/NEBcutter2/?noredir), and possible signal peptides in the gene were analyzed using SignalP-6.0 web tool (https://services.healthtech.dtu.dk/services/SignalP-6.0/). The possibility that the designed primers form dimers within and between themselves was evaluated using the Thermo Fisher Scientific Multiple Primer Analyzer (https://www.thermofisher.com). Melting temperatures were calculated using the NEB Tm Calculator web tool, considering the DNA polymerase to be used in the PCR reaction.

Genomic DNA from cells recovered by centrifuging an overnight fresh culture of *P. sivasensis* R11S16 at 10,000 x *g* for 10 min was extracted using the Invitrogen™ PureLink™ Genomic DNA Mini Kit following the manufacturer’s guidelines. YqiA/YcfP family alpha/beta fold hydrolase gene was amplified using forward (5′-ATT*CTCGAG*CTGTATATCCACGGTTTC-3′) and reverse (5′-AAT*CTCGAG*CAGTGCCGAAAAATC-3′) primers. Amplification was performed in a thermocycler microtube containing 10X Taq Buffer (3 µL), Taq DNA Polymerase (0.25µL: 1.25 U), genomic DNA (3 µL), 10 µmol forward primer (0.8 µL), 10 µmol reverse primer (0.8 µL), 10 mM dNTP (0.8 µL), and nuclease-free water (18.95 µL) (Adıgüzel 2020). The reaction mixture was subjected to pre-denaturation in a thermal cycler (Biorad- T100™, Hercules, CA) at 95 °C for 2 min, preceded by 30 cycles: 40 s denaturation at 95 °C, 40 s oligonucleotide annealing at 60 °C, and 60 s extension at 72 °C, followed by a final extension step at 72 °C for 5 min. Plasmid pET20b(+) was extracted from *E. coli* DH5α cells using FavorPrep™ Plasmid Extraction Kit (Favorgen, Pintung, Taiwan). PCR product and pET20b(+) were separately digested with XhoI (# ER0691) according to the manufacturer’s user guide. After the resulting digests were extracted from 0.8% agarose gel using the GeneJET Gel Extraction Kit, 6 µL of the PCR product and 2 µL of linearized pET20b(+) were ligated by incubating the reaction mixture composed by adding 1 µL of 10X T4 DNA ligase buffer, 0.5 µL of 5U/µL T4 DNA ligase (# 15224041) and 0.5 µL of nuclease-free water at 25 °C overnight (Tabor 1989). DNAs in the ligation mixture (3.5 µL) were introduced into CaCl_2_-competent *E. coli* DH5α cells (50 µL) using the heat-shock transformation protocol described by Chung and Miller (1993). Transformants were grown on LB Agar medium supplemented with ampicillin to a final concentration of 50 µg/mL, followed by agarose gel electrophoresis of plasmids rapidly extracted from replicates of transformants, and clones containing the recombinant molecule were identified by comparing their lengths (Choudhary and Laurie 1991). Afterward, the verified recombinant molecule was subcloned into *E. coli* BL21(DE3) using the same transformation method for heterologous expression of the gene.

### Heterologous expression and purification

A loop of clones containing the recombinant molecule was transferred to 50 mL of LB liquid medium containing ampicillin in a 250 mL Erlenmeyer flask and cultured at 37 °C, 175 rpm with shaking until the optical density (OD_600_) reached 0.4–0.6. IPTG was then added to the culture to a final concentration of 0.3 mM, and the culture was further incubated for 5 h at 18 °C and 175 rpm under shaking conditions (Adigüzel et al. 2023). After incubation, the culture was centrifuged at 10,000 x *g* for 5 min, and the recovered supernatant was treated as a crude enzyme solution. Expression was verified by sodium dodecyl sulfate-polyacrylamide gel electrophoresis (SDS-PAGE) and esterase assay.

The crude enzyme solution was concentrated by passing through a 10-kDa molecular weight cut-off (MWCO) ultrafiltration disk fabricated with regenerated cellulose (Ultracell^®^, Merck Millipore, MA, USA) utilizing an Amicon™-Stirred Ultrafiltration Cell (Merck KGaA, Darmstadt, Germany) (Cilmeli et al. 2024). A. Equal volumes of concentrated crude enzyme solution and HisPur™ Ni-NTA resin (Thermo Scientific™) were gently mixed and kept at 4 °C for 1 h. Unbound proteins were washed through the column with phosphate buffer (50 mM, pH 7.4), and then bound proteins were eluted with a linear gradient of 100–400 mM imidazole prepared in washing buffer at a flow rate of 0.3 mL/min (Adıgüzel et al. 2024). Fractions (each of 1 mL) exhibiting esterase activity were then pooled, concentrated, and dialyzed against 50 mM phosphate buffer (pH 7.0) for further studies.

### SDS-PAGE analysis, total protein estimation and esterase assay

SDS-PAGE analysis was conducted on a 10% acrylamide gel using a modified version of the method developed by Laemmli (1970). Following electrophoresis performed with Mini-Protean Tetra Cell Electrophoresis System (Bio-Rad Laboratories, Hercules, CA), protein bands visualized by Coomassie staining (41) (Adigüzel and Tunçer 2016) were imaged using the Gel Doc XR System (Bio-Rad Laboratories, Hercules, CA). Total protein concentrations of the enzyme solutions were estimated via the method outlined by Bradford (1976) using a bovine serum albumin (BSA) standard curve. Esterase activity was assayed by spectrophotometric (410 nm) monitoring of the release of the chromophore *p*-nitrophenolate (*p*-NP) from the chromogenic substrate *p*-nitrophenyl butyrate (*p*-NPB) as previously described by Adıgüzel (2020).

### Bioinformatic analysis

The cloned YqiA/YcfP family alpha/beta fold hydrolase gene from *P. sivasensis* R11S16 was sequenced at BM Labosis (Ankara, Turkey) using Sanger sequencing and subsequently aligned with the homolog in the type strain of *P. sivasensis* using the Clustal W algorithm from BioEdit software 7.2.5 (https://itservices.cas.unt.edu/software/bioedit725). The aligned genes were visualized in Jalview. The possible functional and structural domains of the enzyme encoded by the gene were identified using the PFAM database, which performs searches based on the HMMER algorithm (Paysan-Lafosse et al. 2025). Physicochemical features of the enzyme expressed in *E. coli* BL21(DE3) were calculated using the ExPASy ProtParam tool (Gasteiger et al. 2005). The secondary (2D) structure of the expressed enzyme was investigated using the “SAS-Sequence Annotated by Structure” tool (Milburn et al. 1998), while its tertiary structure was built using the SWISS-MODEL web server (Waterhouse et al. 2018).

### Biochemical characterization of PsEst_yfh_

The effect of temperature was examined by monitoring enzyme activity over a temperature range of 5–50 °C in 50 mM Tris-HCl buffer (pH 8). The influence of pH on PsEst_yfh_’s activity was studied by performing esterase assays within a pH range of 4 to 10 at optimum temperature, utilizing citrate (pH 4–6), phosphate (pH 6–8), and Tris–HCl (pH 8–10) as buffer systems (50 mM). For each experiment, results are expressed as percentages based on the highest activities of the enzyme, defined as 100% (Adıgüzel et al. 2025).

The stability of PsEst_yfh_ was assessed by measuring the esterase activity after pre-incubation of the enzyme at 30 and 40 °C for up to 6 h. The initial esterase activity was accepted 100%. The enzyme solution prepared in the above-mentioned buffer systems of different pHs was pre-incubated for 1 h at 20 °C, and then the percentage residual esterase activity was determined under optimal conditions to assess the effect of pH on PsEstyfh’s stability (Adigüzel and Tunçer 2016).

The effect of NaCl on PsEst_yfh_ was investigated by examining esterase activity in the presence of NaCl at concentrations up to 16%. The maximum esterase activity measured was assumed to be 100% (Adıgüzel et al. 2024).

The influence of metal ions (Ni^2+^, Ca^+ 2^, Co^+ 2^, K^+^, Mg^+ 2^, Cu^+ 2^, and Fe^+ 2^) and chemicals (βME, EDTA, SDS, Triton X-100, and Tween 20) was evaluated by assaying the esterase activity of PsEst_yfh_ in conditions where the final concentrations of these ingredients were 1 and 10 mM. The results are presented as relative activity in percent compared to the esterolytic activity of PsEst_yfh_ assayed under the same conditions without metal ions or chemicals (100%) (Adıgüzel et al. 2024).

To determine the effects of ethanol, acetone, methanol, dimethyl sulfoxide (DMSO), chloroform, 2-propanol, and butanol, the esterolytic activity of the enzyme was measured under optimal conditions in the presence of 10% v/v or 30% of the solvents. The data were expressed as relative activity, as a percentage of esterase activity measured in the solvent-free reaction mixture under the same conditions (Adigüzel and Tunçer 2016).

The catalytic activity of PsEst_yfh_ against *p*-nitrophenyl acetate (*p*-NPA; C_2_), *p*-nitrophenyl butyrate (*p*-NPB; C_4_), *p*-nitrophenyl caprylate (*p*-NPC; C_8_), *p*-nitrophenyl decanoate (*p*-NPD; C_10_), *p*-nitrophenyl laurate (*p*-NPL; C_12_), *p*-nitrophenyl myristate (*p*-NPM; C_14_), and *p*-nitrophenyl palmitate (*p*-NPP; C_16_) was determined through the standard assay method under optimum conditions to assess the substrate specificity. The highest catalytic activity was considered to be 100% (Adıgüzel 2020).

The enzyme’s catalytic efficiency against *p*-NPB was evaluated based on the Michaelis–Menten constant (*K*_*m*_) and maximum velocity (*V*_*max*_) values calculated from the Lineweaver-Burk plot, which was constructed from enzyme activities in reaction mixtures containing substrate at concentrations ranging up to 4 mM. Enzymatic reactions were performed in 50 mM Tris-HCl buffer at 20 °C (Adıgüzel et al. 2025).

### Molecular docking

The binding interaction between PsEst_yfh_ and substrates was examined using molecular docking, as previously reported by Adıgüzel et al. (2025). PsEst_yfh_’s 3D structure in pdb format, constructed using the Swiss Model, was used as a receptor. The 3D molecular structures of the substrates were retrieved from the PubChem database (https://pubchem.ncbi.nlm.nih.gov/) in SDF format. Substrates and receptors were prepared for docking by performing dehydration, charge neutralization, and hydrogenation using UCSF Chimera 1.18 software (Pettersen et al. 2004). Afterward, molecular docking was performed within a grid box encompassing the entire PsEst_yfh_ structure using AutoDock Vina v1.1.2 (Trott and Olson 2010). The exhaustiveness parameter was set to 8, and a total of 10 binding modes were generated for each ligand with a maximum energy range of 3 kcal/mol. Finally, the enzyme-substrate combinations with the lowest binding free energies were selected, and their interactions were analyzed using BIOVIA Discovery Studio Visualizer 2025 software (Dassault Systèmes).

### Removal of azithromycin antimicrobial activity

The experiments were performed as described by Adıgüzel et al. (2023), with minor modifications. The reactions were carried out at 20 °C for 120 min in a 50 mM Tris-HCl buffer (pH 9) supplemented with 50 µg/mL of azithromycin and 20 U/mL of PsEst_yfh_. The removal of antimicrobial activity was monitored by sampling at 20, 40, 60, 90, and 120 min. The enzyme in the reaction mixture was removed by ultrafiltration. The resulting enzyme-free solutions were passed through sterile syringe filters and analyzed for residual antimicrobial activity against *Bacillus subtilis* using the agar well diffusion method (Mazmancı et al. 2023). To that end, ~ 5 × 10⁷ bacterial cells were spread on Mueller-Hinton Agar (MHA) plates, then wells with a diameter of 8 mm were created. Afterward, 70 µL from each sample was poured into the wells. The plates were incubated at 37 °C for 16 h, and the antimicrobial activity was determined by measuring the diameters of the inhibition zones. Time-dependent changes in zone size were used to assess the enzyme’s effect on azithromycin’s antimicrobial activity of azithromycin. An enzyme-free reaction mixture was used as a positive control. The percent decrease in the antibiotic’s antimicrobial activity was calculated using the following formula (1).

1 $$\begin{gathered} Decrease~in~antimicrobial~activity~\left( \% \right) = \left[ {100 - \frac{{\left( {E - 50.24} \right)~ \times ~100}}{{T_{0} }}} \right] \hfill \\ - \left[ {100 - \frac{{\left( {T_{4} - 50.24} \right)~~ \times ~100}}{{T_{0} }}} \right] \hfill \\ \end{gathered}$$

where, $\:{T}_{0}$ represents the inhibition zone area of the antibiotic solution, $\:E$ corresponds to the inhibition zone area measured for the reaction mixture following enzymatic treatment, and $\:{T}_{4}\:$ denotes the inhibition zone area of the enzyme-free reaction control. The surface area of the agar well was calculated to be 50.24.

For kinetic evaluation, the decrease in antimicrobial activity was analyzed under the assumption of pseudo-first-order kinetics by calculating − ln(Aₜ/A₀) values from the remaining activity data.

### Ecotoxicological assessment

The acute toxicity of the end-products after removal of the azithromycin antimicrobial activity was evaluated using duckweed (*Lemna minor*) as the test organism (Radić et al. 2010). After the removal, the reaction mixture was passed through a 10 kDa MWCO ultrafiltration membrane. The ultrafiltrate, containing the end products after removal, was used as a test group for toxicological evaluation, while the buffer solution used to prepare the reaction mixture served as a control group.

*L. minor* was obtained from a commercial firm and subjected to 2 month of acclimaton in 500 ml glass beakers containinng 100 mL in 10% the original Hoagland’s nutrient medium under a 16:8 h light: dark period of fluorescent light with a light intensity of 6500–10,000 lx at 24 ± 2 °C (Fekete-Kertesz et al. 2015; Zhou et al. 2020). Stock cultures were transferred to fresh medium in every 3–7 days. Healthy duckweeds with two frond colonies were placed in 15 mL-petri dishes containing 10 mL of the test and control groups. The toxicity test was conducted by exposing duckweeds to the control and test groups for 7 days. Toxicity was recorded as growth parameters (number of fronds), fresh weight, dry weight, and photosynthetic pigment content (chlorophyll a, chlorophyll b, and carotenoids).

Duckweed growth was determined by measuring frond number, fresh weight, and dry weight, according to the ISO 20,079 test protocol (Radić et al. 2010). The frond number was scored at the start (T_0_) of the experiment and 7 days (T_7_) after. All visible fronds were counted. To measure fresh weight, *L. minor* was surface-dried between layers of paper towels. Growth was determined as Relative Growth Rate (RGR, g/days) as follows; log N final fresh weight (g) – log N initial fresh weight (g)/ time (days) (Basiglini et al. 2018). The dry weight was determined after plants were dried at 80 °C for 24 h to calculate dry weight (g) to fresh weight (g) ratio (DW/FW) (Radić et al. 2011). To assess the potential toxic effect of the tested materials, the Percent Inhibition of Growth Rate ($\:\mathrm{\%}{I}_{r}$) was calculated based on the number of frond measurements according to the formula (2) given below (OECD 2006):

2 $$\:\mathrm{\%}{I}_{r}=\left[\frac{\left(\mu\:c-\mu\:r\right)\:}{\mu\:c}\:\times\:\:100\right]\:\:\:\:$$

where, $\:\mu\:c$ and $\:\mu\:r$ are the average number of fronds in the control group and test group, respectively.

The photosynthetic pigment content of duckweeds is widely used to evaluate the effect of a xenobiotic on the photosynthetic system. Fronds weighing 0.2 g were picked from each treatment and control group on the 7th day of the experiment to determine pigment content. Fronds were homogenized with 80% cold acetone (1:15 w/v) to prepare the extract and, the obtained homogenate was then centrifuged at 8000 x rpm for 10 min at 4 °C (Basiglini et al. 2018). The supernatant was collected into clean tubes and analyzed by spectrometry. To determine the chlorophyll and carotenoid content, optical densities at 461, 646.8, and 663.2 nm wavelengths were measured using a UV-visible spectrometer. Then, chlorophyll a (Chl a), chlorophyll b (Chl b), total chlorophyll (Chl tot), and total carotenoid (TC) contents were calculated using the following formulas (3–6) (Kumar et al. 2024).

3 $$\:\mathrm{C}\mathrm{h}\mathrm{l}\:\mathrm{a}\:({\upmu\:}\mathrm{g}/\mathrm{m}\mathrm{L})=\left[\left(12.25\:\times\:\:{OD}_{663.2}\right)-\left(2.79\:\times\:\:{OD}_{646.8}\right)\right]\:$$

4 $$\:\mathrm{C}\mathrm{h}\mathrm{l}\:\mathrm{b}\:({\upmu\:}\mathrm{g}/\mathrm{m}\mathrm{L})=\left[\left(21.50\:\times\:\:{OD}_{646.8}\right)-\left(5.10\:\times\:\:{OD}_{663.2}\right)\right]$$

5 $$\:\mathrm{C}\mathrm{h}\mathrm{l}\:\mathrm{t}\mathrm{o}\mathrm{t}\:({\upmu\:}\mathrm{g}/\mathrm{m}\mathrm{L})=\left[\left(7.15\:\times\:\:{OD}_{663.2}\right)+\left(18.71\:\times\:\:{OD}_{646.8}\right)\right]$$

6 $$\:\mathrm{T}\mathrm{C}\:({\upmu\:}\mathrm{g}/\mathrm{m}\mathrm{L})=\left[{OD}_{461}-\left(0.046\:\times\:\:{OD}_{663.2}\right)\right]\times\:4$$

### Statistical analysis

All experiments, except for the 16 S rRNA-based identification of the bacterial strain and the cloning of the YqiA/YcfP family alpha/beta fold hydrolase gene, were performed in triplicate. Results were expressed as mean ± standard deviation (SD) after being computed using Microsoft Excel Software. The results are also plotted on the graph with standard error bars. Statistical significance of data from characterization and azithromycin removal experiments was evaluated using one-way analysis of variance (ANOVA), followed by pairwise comparisons using Tukey’s post hoc test. Results from the experiment, performed for the ecotoxicological assessment, were analyzed by a t-test. Sigma Plot 11 (Systat Software, USA) was used for all statistical analyses.

## Results and discussion

### Phylogenetic affiliation of strain R11S16

**Fig. 1:**
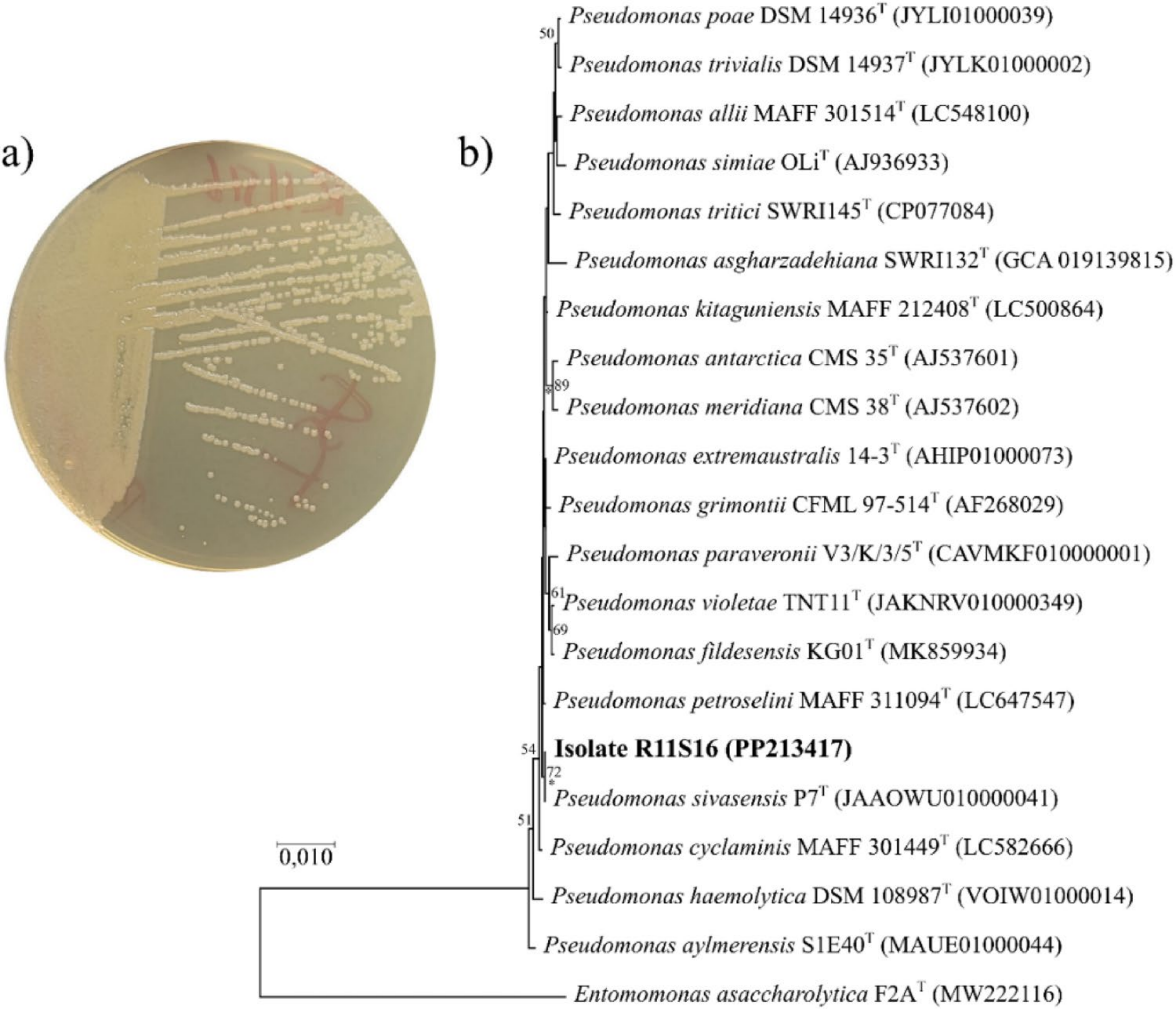
Phylogenetic affiliation of the enzyme-producing strain R11S16. **a** Colony morphology of strain R11S16 grown on TSA following incubation at 15 °C for three days. **b** Neighbor-joining phylogenetic tree based on 16 S rRNA gene sequences showing the position of strain R11S16 among closely related *Pseudomonas* type strains. Bootstrap values (> 50%) based on 1000 replications are shown at branch nodes. Nodes marked with an asterisk (*) indicate clades that were consistently supported by MP and ML trees. *Entomomonas asaccharolytica* F2A^T^ was used as the outgroup. Bar, 0.010 substitutions per nucleotide position

The nearly complete 16 S rRNA gene sequence (1459 bp) of strain R11S16 was obtained. Comparative sequence analysis revealed that strain R11S16 showed 100.0% sequence similarity to *Pseudomonas sivasensis* P7^T^, followed by *Pseudomonas petroselini* MAFF 311,094^T^ (99.93%) and *Pseudomonas extremaustralis* 14-3^T^ (99.86%) (Table 1). Phylogenetic analysis based on 16 S rRNA gene sequences placed strain R11S16 within the genus *Pseudomonas*, clustering tightly with *P. sivasensis* P7^T^ (Fig. 1). Comparable tree topologies indicated a stable and consistent phylogenetic placement of strain R11S16. Although several closely related *Pseudomonas* species exhibited high 16 S rRNA gene sequence similarity, strain R11S16 consistently grouped with the type strains of *P. sivasensis* lineage with strong bootstrap support across all phylogenetic reconstructions. Strain R11S16 formed smooth, circular, cream-colored colonies on Tryptic Soy Agar (Fig. 1), consistent with phenotypic characteristics commonly reported for members of the genus *Pseudomonas* (Méndez et al., 2017). Notably, several phylogenetically related species included in the analysis have previously been reported from cold or temperate environments, supporting the ecological relevance of strain R11S16 as a psychrotolerant bacterium and a potential source of cold-active enzymes (Duman et al. 2020; Xiong et al. 2023). Based on 16 S rRNA gene sequence similarity and phylogenetic clustering with the type strain, strain R11S16 was assigned to *P. sivasensis*. This level of molecular identification was considered sufficient to provide a reliable taxonomic framework for subsequent analyses focusing on the biochemical characterization and industrial potential of the cold-active esterase produced by strain R11S16.

**Table 1:** Pairwise 16 S rRNA gene sequence similarities between strain R11S16 and the most closely related *Pseudomonas* type strains

Most closely related type strain	Accession number	Similarity (%)	nt differences
*Pseudomonas sivasensis* P7^T^	JAAOWU010000041	100.00	0/1459
*Pseudomonas petroselini* MAFF 311,094^T^	LC647547	99.93	1/1459
*Pseudomonas extremaustralis* 14-3^T^	AHIP01000073	99.86	2/1459
*Pseudomonas cyclaminis* MAFF 301,449^T^	LC582666	99.86	2/1459
*Pseudomonas kitagugiensis* MAFF 212,408^T^	LC500864	99.86	2/1459

### Cloning, heterologous expression and purification

The gene YqiA/YcfP family alpha/beta fold hydrolase (*yfh*) amplified by PCR from the genomic DNA of *P. sivasesis* R11516 isolate was analyzed electrophoretically, and a single DNA band of about 600 bases was observed on 0.8% agarose as predicted (Supplementary information 1). The amplified yfh gene and pET20b(+) were successfully digested with XhoI, ligated, and the thus generated construct (pET20b(+)-*yfh*) was transformed into *E. coli* DH5α (Supplementary information 2). Transformants grown overnight at 37 °C in the presence of ampicillin were screened as harboring the pET20b(+)-*yfh* by comparing the size of the rapidly isolated plasmids (Supplementary information 3). The insert in the recombinant molecule was identified by sequencing using the universal T7 promoter (5′- TAATACGACTCACTATAGGG-3′) and T7 terminator (5′-GCTAGTTATTGCTCAGCGG-3′) primers. Thus, recombinant molecule and the gene orientation was verified. Furthermore, the cloned gene was aligned with its homolog in the type strain *P. sivasensis* using Clustal Omega (https://www.ebi.ac.uk/jdispatcher/msa) and visualized in Jalview (Waterhouse et al. 2009) (Supplementary information 4). Accordingly, the 108th, 254th, 256th, 257th, 275th, and 297th nucleotides in the *yfh* gene of isolate R11516 were found to be different from those of the type strain of *P. sivansensis*. As a result of protein-based analyses performed on NCBI (https://blast.ncbi.nlm.nih.gov/Blast.cgi) database; the amino acid content of the gene product was found to be different YqiA/YcfP family alpha/beta hydrolases from *P. sivasensis* (Protein accession number: WP_181640331.1), *Pseudomonas cyclaminis* (Protein accession number: WP_122305492.1), *Pseudomonas fluorescens* (Protein accession number: WP_191946927. 1), *Pseudomonas grimontii* (Protein accession number: WP_090400878.1), *Pseudomonas rhodesiae* (Protein accession number: WP_348749120.1) and *Pseudomonas azotoformans* (Protein accession number: WP_071497189. 1) by 97.0%, 96.5%, 95.5%, 95.5%, 95.0%, 94.5% and 94.0%, respectively. Finally, the verified recombinant molecule was subcloned into *E. coli* BL21(DE3).

**Fig. 2:**
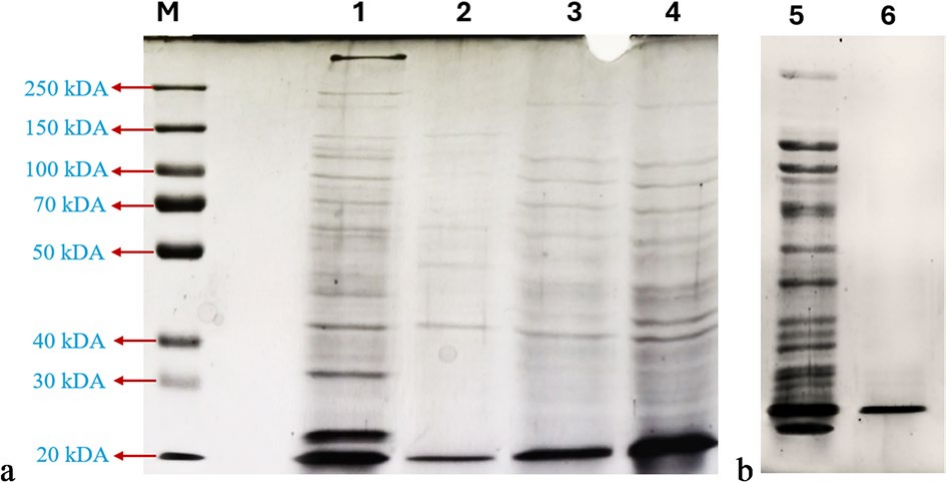
**a** SDS-PAGE analysis showing the extracellular protein profiles of (Line 1) *E. coli* BL21(DE3) cells harboring pET20b(+)-yfh in the presence of IPTG, (Line 2) *E. coli* BL21(DE3) cells harboring pET20b(+) in the presence of IPTG, (Line 3) *E. coli* BL21(DE3) cells in the absence of IPTG, (Line 4) *E. coli* BL21(DE3) cells harboring pET20b(+) in the presence of IPTG. Line M indicates the protein marker. **b** SDS-PAGE analysis of the (Line 5) crude enzyme solution and (Line 6) enzyme solution after purification

The gene *yfh* was extracellularly expressed with a histidine tag (6X-His) under the control of the T7 promoter. Expression of the gene was assessed by SDS-PAGE analysis (Fig. 2a). On the acrylamide gel, the expression of *yfh* gene was proved by the observation of a unique protein band with a molecular weight of approximately 23 kDa in the extracellular liquid from the culture of *E. coli* BL21(DE3) cells harboring pET20b(+)-yfh, in comparison with the extracellular liquid from the culture of *E. coli* BL21(DE3) cells with and without pET20b(+). In addition, the esterase titer in the crude enzyme solution recovered from the IPTG-induced culture of the *E. coli* BL21(DE3) harboring the pET20b(+)-yfh was 56.83 ± 0.82 U/mL. In contrast, no extracellular esterase activity was detected in the culture of *E. coli* BL21(DE3) harboring pET20b(+) grown under the same conditions. The expressed recombinant protein was designated PsEst_yfh_. The specific activity of PsEst_yfh_ was 170.10 ± 5.09 U/mg protein. SDS-PAGE analysis also revealed that the enzyme had a lower molecular weight than the cold-active esterases from *Pseudomonas* sp. TB11 (Dong et al., 2015), *Serratia* sp. (Jiang et al., 2016), *Zunongwangia profunda* (Rahman et al., 2016), *Bacillus halodurans* (Noby et al., 2020), *Pseudomonas* sp. E2-15 (Liu et al., 2021), *Deinococcus radiodurans* (Zhang et al., 2021), *Pseudomonas* sp. E5-12 (Liu et al., 2022), *Aphanizomenon flos-aquae* (Knepp et al., 2022), *Arthrobacter soli* (Cao et al., 2023), *Halobacterium salinarum* (Ortega-de la Rosa et al., 2024), *Haloarcula japonica* (Kato et al., 2025), and *Aspergillus niger* (Xing et al., 2025). On the other hand, it had a similar molecular weight to cold active esterases from *Psychrobacter pacificensis* (Wu et al., 2013), *Bacillus cohnii* (Noby et al., 2018), and *Lysinibacillus* sp. (Matrawy et al., 2022). In summary, PsEst_yfh_ may be the smallest cold-active esterolytic enzyme ever reported to our knowledge. PsEst_yfh_’s small nature makes it worthwhile for fusion protein construction with other cold-active enzymes, notably laccase, for bioremediation.

### Bioinformatic analysis of PsEst_yfh_

Protparam analysis of the expressed enzyme confirmed its molecular weight of about 23024.75 Da. The analysis revealed that the enzyme has an isoelectric point of 6.11 and a half-life of more than 10 h in *E. coli*. The instability value of PsEst_yfh_, calculated as 37.26, indicates that it is stable. The aliphatic index value of the enzyme, based on the ratio of aliphatic (branched chain) amino acids (alanine, valine, isoleucine, and leucine) in its structure, was found to be 86.01. The detected Grand average of hydropathicity (GRAVY) value for the enzyme was negative (-0.336), indicating that the enzyme is mainly water-soluble.

**Fig. 3:**
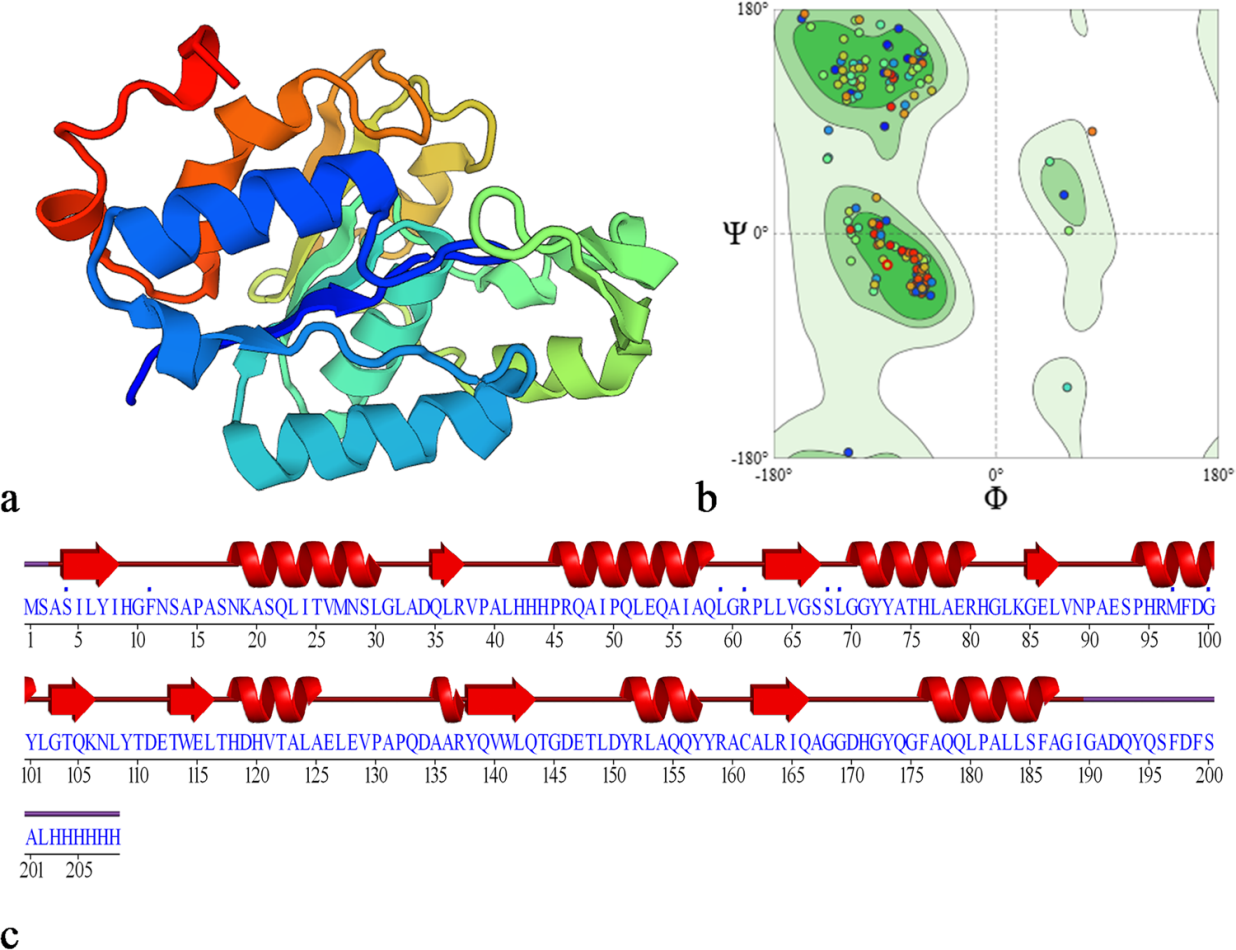
**a** PsEst_yfh_’s 3D structure constructed by SwissModel. Blue and red regions represent the N-terminus and C-terminus, respectively. **b** Ramachandran plot of φ, ψ torsion angles of amino acid residues in PsEst_yfh_. **c** Secondary structure analysis of PsEst_yfh_. Serin: Ser/S, Aspartic acid: Asp/D, Histidine: His/H. Flat arrows are a simplified representation of β-sheets, and the direction of the arrow indicates the direction of the amino acid sequence (from the N-terminus to the C-terminus). Ribbons depict α-helix structures. Thin, straight lines indicate regions lacking secondary structure

The 3D structure of PsEstyfh was generated by the SwissModel web tool based on the *Pseudomonas trivialis* esterase deposited in the Protein Data Bank (PDB) with the code A0A0H5AAT2.1.A (Fig. 3a). The amino acid sequence of PsEst_yfh_ was 93.50% identical to that of *P. trivialis* esterase. The GMQE (Global Model Quality Estimate) for the modeled enzyme was 0.88, indicating the model’s reliability. The MolProbity score of the model was computed as 0.73 based on the Ramachandran plot shown in Fig. 3b. The Ramachandran Favored value (97.47%) indicated that the enzyme folded into common and sterically allowed angle combinations in natural proteins. Ramachandran Outliers and Rotamer Outliers values, which indicate the rate of significant conformational errors, were found to be 0%. A topological analysis proves that PsEst_yfh_ exhibits a classical α/β fold. The analysis performed on the secondary structure of PsEst_yfh_ revealed that the enzyme comprises 8 alpha helices and 7 beta-sheets (Fig. 3c). It is also observed that the conserved Gly-X-Ser-X-Gly pentapeptide motif is positioned at the end of the 3rd beta-sheets, while Ser-68, Asp-146, and His-171, forming the catalytic triad, are located after the 3rd, 7th, and 8th beta-sheets, respectively.

### Biochemical characterization of PsEst_yfh_

**Fig. 4:**
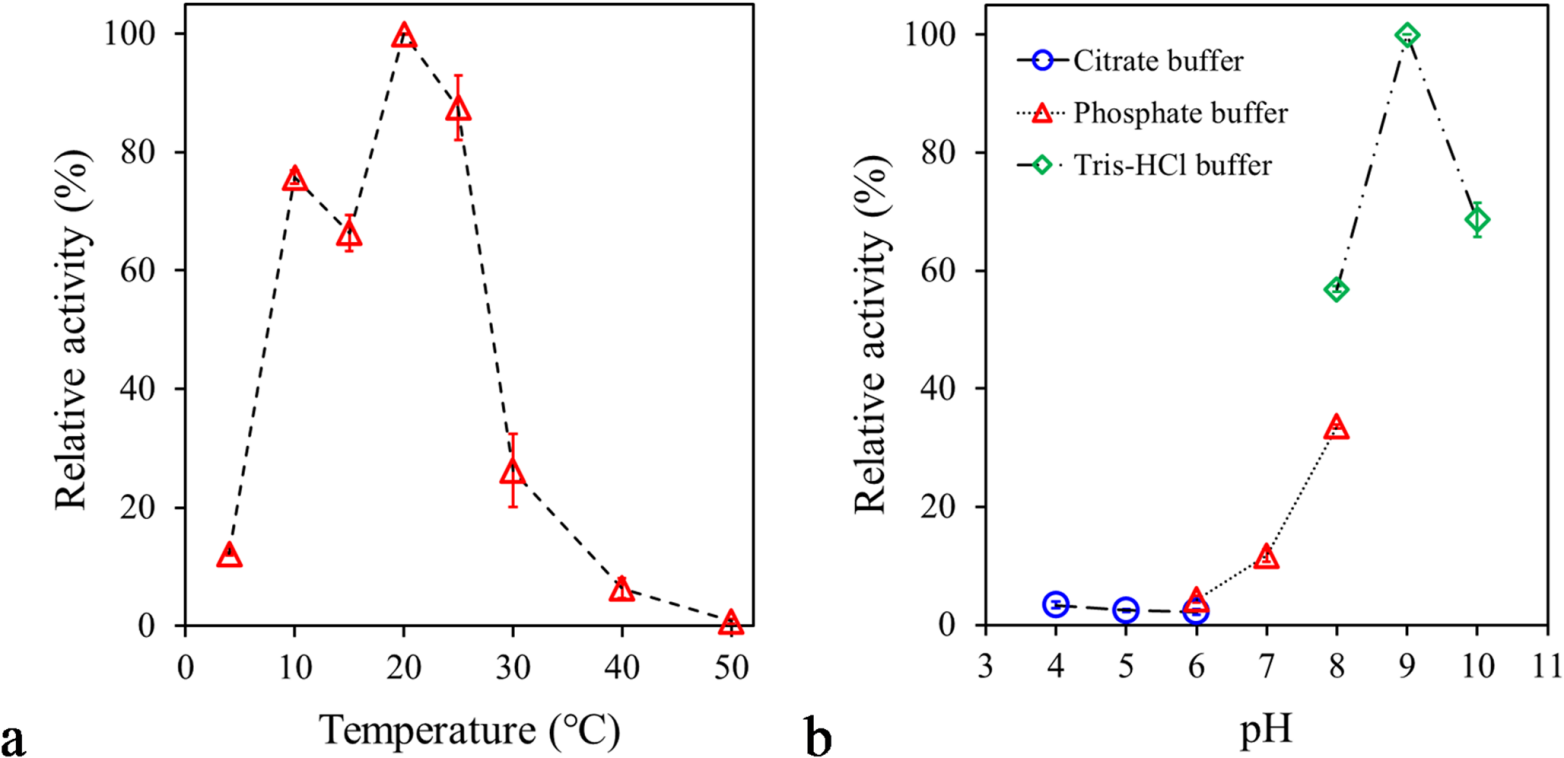
**a** The influence of temperature (5–50 °C) on esterolytic activity of PsEst_yfh_. **b** Effect of pH on the activity of PsEst_yfh_. Citrate (blue empty circles), phosphate (red empty triangles), and Tris–HCl (green empty diamonds) buffer systems were used to adjust the pH of the reaction mixtures. The highest esterase activity for each set of experiments was regarded as 100%. Results from triplicate experiments were displayed, with bars representing the standard error

The experiments to evaluate the effect of temperature (5–50 °C) revealed that PsEst_yfh_ exhibited a distinct temperature optimum at 20 °C (Fig. 4a). At 4 °C, the enzyme retained only 12.21 ± 0.46% of its maximal activity (*P < 0.001*), whereas its activity increased markedly to 75.80 ± 1.98% at 10 °C (*P < 0.001*). Such a substantial increase in activity between 4 °C and 10 °C indicates that the enzyme requires a minimum threshold of thermal energy for efficient catalysis. This behavior suggests a partially psychrophilic character but indicates that the enzyme may not be fully active in extreme cold environments. PsEst_yfh_ retained 87.54% activity at 25 °C, indicating a reasonable activity beyond its optimum (*P < 0.05*). However, at 30 °C, the enzyme’s relative activity decreased to 26.30% (*P < 0.001*). A further substantial decrease was observed by increasing the temperature to 40 °C and 50 °C, remaining at 6.40 ± 2.82% and 0.84 ± 0.74% relative activity, respectively. These findings suggest the significant thermal variability typical of enzymes adapted to low-temperature conditions; the increased flexibility of the enzyme’s structure increases catalytic efficiency at low temperatures while making it susceptible to thermal denaturation at high temperatures. A similar phenomenon was noted for esterases exhibiting maximum activity at temperatures of 0–30 °C, such as those from *Lactobacillus plantarum* (Esteban-Torres et al., 2014), *Glaciozyma antarctica* (Hashim et al., 2018), *Bacillus cohnii* N1 (Noby et al., 2021), *D. radiodurans* (Zhang et al., 2021), *A. flos-aquae* (Knepp et al., 2022), *Marinomonas* sp. ef1 (Marchetti et al., 2023), *Pseudomonas* sp. TB11 (Sha et al., 2024). In addition, PsEst_yfh_’s small size may have contributed to its strong sensitivity to temperatures above 25 °C. On the other hand, in contrast to PsEst_yfh_, many cold-active esterases, such as those from EstC (Kato et al. 2025), EstS9N (Wicka et al. 2016), MtEst45 (Lee 2016), Est A (Ke et al. 2018), Est19 (Liu et al. 2021), EstRag (Matrawy et al. 2022), and Est1260 (Liu et al. 2023) have been reported to exhibit maximum activity above 30 °C. Ultimately, the enzyme’s behavior at the tested temperatures could be advantageous in processes requiring catalysis at low to medium temperatures, contributing to energy savings and preserving heat-sensitive substrates.

The cold-active properties of PsEst_yfh_ can be attributed to several structural adaptations within its amino acid sequence. The maintenance of catalytic efficiency at low temperatures may be associated with a hydrophobic residue ratio of about 40%, which supports structural flexibility. It can also be explained within the framework of the activity-flexibility-stability trade-off characteristic of cold-adapted enzymes, which enables the protein to undergo the necessary conformational changes for catalytic activity at low temperatures (Dhaulaniya et al. 2019). Furthermore, the relatively high glycine content (~ 9%) enhances backbone mobility due to its lack of a side chain, thereby lowering the activation energy barrier and increasing efficiency at low temperatures (Truongvan et al. 2016). The abundance of uncharged polar residues, such as glutamine and threonine, further contributes to this structural flexibility (Kashif et al. 2017).

The activity of PsEst_yfh_ was investigated across a range of pH values using citrate (pH 4–6), phosphate (pH 6–8), and Tris-HCl (pH 8–10) buffer systems. The results summarized in Fig. 4b revealed that PsEst_yfh_’s catalytic activity is highly susceptible to hydrogen ion concentration in the environment. The enzyme showed rather weak catalytic efficiency in acidic conditions, like many other bacterial esterases (He et al. 2024; Yang et al. 2025). The relative esterase activity between pH 4 and pH 6 remained below 5%, and no significant difference was detected among enzyme activities calculated within this pH range (*P > 0.05*). The relative activity of PsEst_yfh_ at pH 7 was calculated to be 11.74 ± 1.79% (*P < 0.001*). PsEst_yfh_ exhibited its highest activity at pH 9. Moreover, the enzyme retained its relatively high catalytic efficiency over a constrained range of alkaline pHs, particularly between pH 8 and 10, indicating a distinct preference for alkaline conditions. Remarkably, PsEst_yfh_ was able to retain more than 65% of its activity at pH 10, unlike its counterparts from *B. halodurans* (Noby et al., 2020), *Pseudomonas* sp. E2-15 (Liu et al., 2021), *D. radiodurans* (Zhang et al., 2021), *Exiguobacterium antarcticum* (Hwang et al., 2023), *Altererythrobacter indicus* DSM 18604 (Li et al., 2025), and *Pseudomonas* sp. D01 (Kuan et al., 2023).

The observed alkaliphilic character of PsEst_yfh_ can be attributed to its specific amino acid composition, a common feature among alkali-stable enzymes. Such enzymes commonly contain more arginine (Arg/R) and neutral hydrophilic residues (His, asparagine/Asn/N, and glutamine/Gln/Q), which are crucial for maintaining structural integrity at high pH. Arginine residues in particular are known to form robust hydrogen bonds and stable ion pairs, thereby neutralizing the destabilizing effects of deprotonation at alkaline pH and helping to preserve the catalytic conformation of the enzyme (He et al. 2024). The fact that 23.1% of the amino acid composition of the enzyme consists of arginine and neutral hydrophilic amino acid residues provides a convincing explanation for the significant activity of the enzyme under alkaline conditions. The combination of the enzyme’s cold-active characteristic and its relatively high catalytic activity in alkaline conditions makes it particularly valuable for various biotechnological applications, including food processing, pharmaceutical synthesis, and bioremediation.

The results also showed that the phosphate buffer system was less favorable than Tris-HCl for enzyme activity. It may be due to the higher ionic strength of phosphate buffer compared to Tris-HCl at the same concentration, which alters the charge distribution in the enzyme’s active site, limiting substrate binding. Alternatively, the cosmotropic phosphate ions may have strong electrostatic interactions with water molecules, forming an ordered hydration shell around the enzyme and thereby reinforcing hydrophobic interactions between amino acids, making it difficult for the active site to undergo the conformational changes required for substrate binding. Another possible reason is the direct inhibitory effect of phosphate ions binding to the active site.

**Fig. 5:**
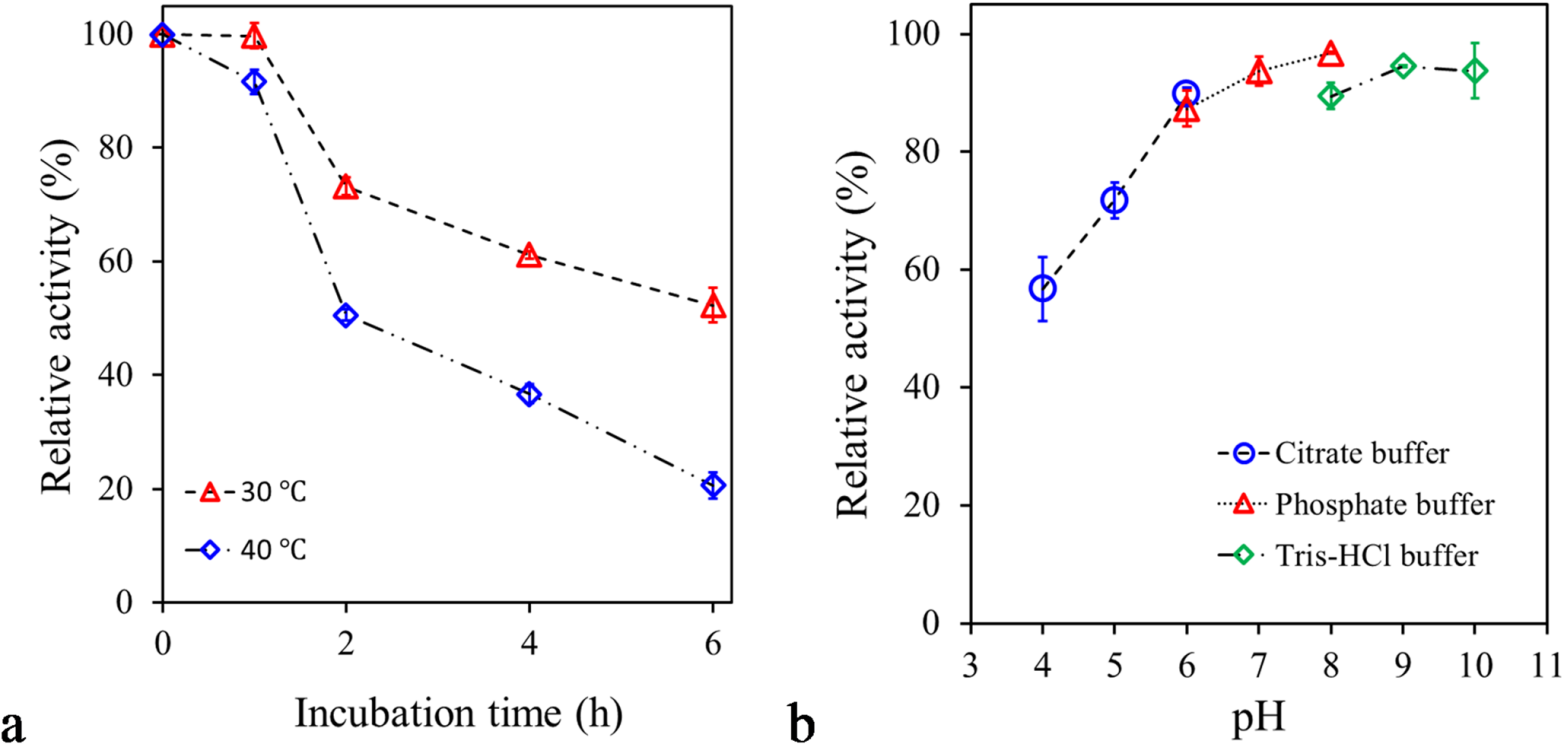
**a** The effect of temperature on stability of PsEst_yfh_. **b** The influence of pH on the stability of PsEst_yfh_. Citrate (blue empty circles), phosphate (red empty triangles), and Tris–HCl (green empty diamonds) were used for adjust pH of the reaction mixture. Experimental data were shown as mean values from triplicate measurements, accompanied by standard error bars

The stability of the enzyme at 30 °C and 40 °C was assessed by pre-incubating the enzyme at these temperatures for 6 h and thereafter calculating its remaining activity (Fig. 5a). After 1 h of pre-incubation at 30 °C, it was found that almost all of the enzyme’s activity was preserved (*P > 0.05*). The relative activity of PsEst_yfh_ was calculated as 73.22 ± 2.76% and 61.19 ± 1.13%, respectively, at the end of 2 and 4 h of pre-incubation at the same temperature (*P < 0.001*). The PsEst_yfh_’s half-life at this temperature was determined to be nearly 6 h. PsEst_yfh_ exhibited moderate stability at 40 °C. It was observed that 91.62 ± 3.61% of the activity was retained after the enzyme was pre-incubated for 1 h under the same conditions (*P < 0.001*). On the other hand, pre-incubation at 40 °C for 2 h reduced the catalytic activity of the enzyme by half. After 4 h and 6 h of pre-incubation at 40 °C, the relative activity of PsEst_yfh_ was 36.72 ± 2.82% and 20.67 ± 3.98%, respectively (*P < 0.001*). Results revealed that PsEst_yfh_ is more stable than various cold-active/adaptive esterases, including EstC (Brault et al. 2012), Est12 (Wu et al. 2013), Est97 (Fu et al. 2013), ThaEst2349 (De Santi et al. 2016), J15 (Shakiba et al. 2016), EstT1-39 (Dong et al. 2017), Est700 (Zhang et al. 2018), EstN7 (Noby et al. 2018), GaDlh (Hashim et al. 2018), and EstRag (Matrawy et al. 2022). The apparent stability of the enzyme under mesophilic conditions increases its robustness to slight temperature variations that may occur during the process, offering additional advantages that will improve the overall flexibility of processes in bioremediation and detergent industry applications.

The stability of PsEst_yfh_ was assessed over the pH range from 4 to 10. The results plotted in Fig. 5b demonstrated that the enzyme was remarkably stable, especially under mildly acidic, neutral, and alkaline conditions. The enzyme exhibited relative activities of 94.53 ± 0.30% and 93.75 ± 8.13% following pre-incubation in Tris-HCl buffers at pH 9 and pH 10, respectively. The enzyme retained 93.67 ± 4.39% and 87.31 ± 5.32% of its initial activity after pre-incubation in phosphate buffers at pH 7 and 6. The change in enzyme activity compared to the initial activity was not statistically significant following pre-incubation between pH 8 and pH 10 (*P > 0.05*). Additionally, pre-incubation in citrate buffer at pH 5 and pH 6 resulted in residual activities of 71.72 ± 5.32% and 89.73 ± 1.94%, respectively, relative to the initial activity (*P < 0.001*). In contrast, a pronounced decrease in stability was observed in citrate buffer at pH 4, where the relative activity dropped to 56.67% following pre-incubation for 60 min at 20 °C (*P < 0.001*). These findings suggest that the enzyme stability markedly declines at more acidic pH values. Even though the Tris-HCl buffer provides a favorable environment for short-term catalytic activity due to its buffering capacity at neutral and slightly alkaline pH conditions, it may not be as effective as the phosphate buffer in maintaining long-term stability. Such a discrepancy may be related to the fact that Tris-HCl leads to weaker ion-protein interactions or the increased structural flexibility of the enzyme in the environment.

**Fig. 6:**
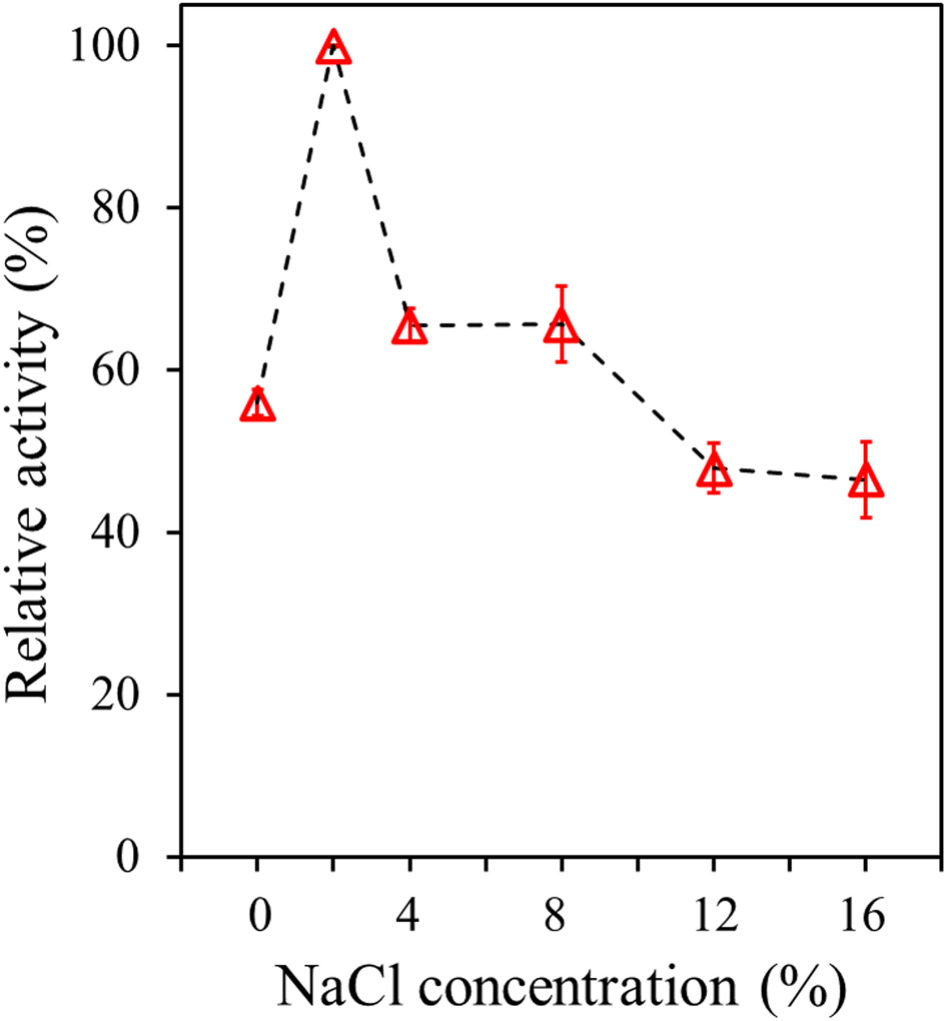
The effect of NaCl on esterolytic activity of PsEst_yfh_. Highest enzyme activity against *p*-NPB accepted as 100%. Data were reported as mean values calculated from triplicate assays, and with standard error bars

The esterolytic activity of PsEst_yfh_ under different NaCl concentrations was examined, revealing that the enzyme exhibited its highest activity at 2% NaCl concentration (Fig. 6). The relative activity of the enzyme in the absence of salt was 55.99 ± 2.67%, which suggested that low salt levels could have exerted a stabilizing effect on the catalytic structure of the enzyme and thus have improved its activity (*P < 0.001*). Although enzyme activity was partially maintained at 4% (65.47 ± 3.70% relative activity) and 8% (65.63 ± 8.09% relative activity) NaCl, a noticeable decline was observed compared to 2% NaCl (*P < 0.001*). At higher salt concentrations (12% and 16%), the decrease in esterolytic activity became more pronounced, indicating that excessive salt may disrupt ionic interactions within the protein structure, negatively impacting the enzyme’s conformational stability and substrate-binding capacity. The results also showed that there was no significant variation in enzyme activity due specifically to increasing NaCl concentrations from 12% to 16% (*P > 0.05*). The findings demonstrate that the PsEst_yfh_ esterase enzyme exhibits moderate NaCl tolerance comparable to that of the cold-active esterases from *Psychrobacter pacificensis* (Wu et al., 2015), Z. *profunda* (Rahman et al., 2016), Siberian permafrost metagenomic DNA library (Boyko et al., 2021), *Lysinibacillus* sp. (Matrawy et al., 2022), *H. salinarum* NCR-1 (Ortega-de la Rosa et al., 2024), but it is less than that of esterases like ESTA92D (Ke et al. 2018) and Est1260 (Liu et al. 2023). A possible reason for PsEst_yfh_’s moderate salt tolerance may be its low lysine content (1.4%) and its greater proportion negatively charged amino acids compared with its mesophilic counterparts (Adıgüzel et al. 2024; Li et al. 2024). Another reason may be that the pI value is in the slightly acidic range (Lu and Daniel  2021).

**Fig. 7:**
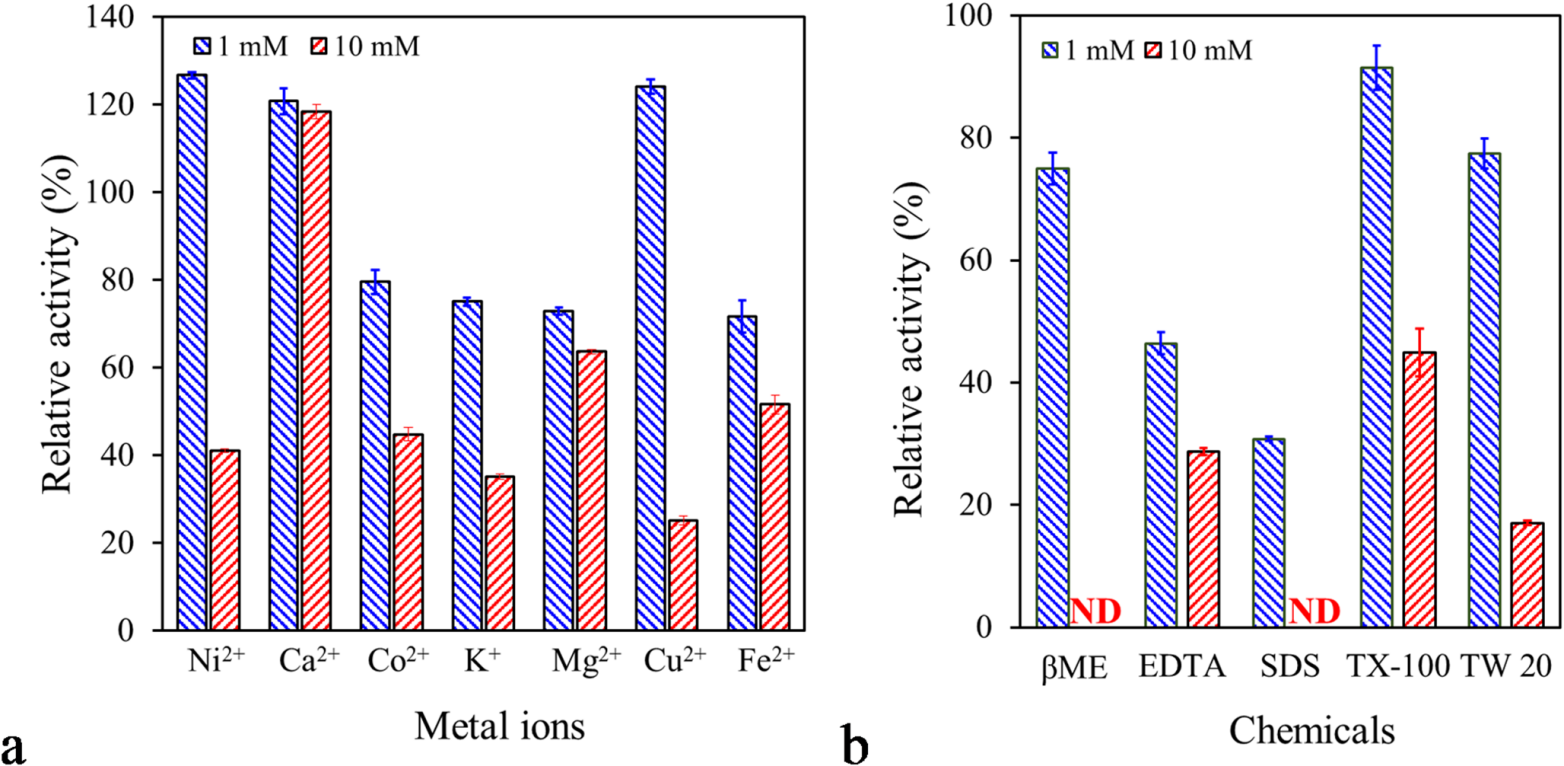
Influence of metal ions (**a**) and various chemicals (**b**) on PsEst_yfh_’s catalytic activity. Enzyme activity was evaluated under optimal assay conditions by incorporating either 1 mM (indicated by filled blue diagonal lines) or 10 mM (indicated by filled red diagonal lines) concentrations of the tested additives. Enzyme activity measured without any additives was defined as the control (100%). All data are represented as the mean of triplicate experiments with standard error bars (βME: β-merkaptoetanol, EDTA: ethylenediaminetetraacetic acid, SDS: sodium dodecyl sulfate, TX-100: Triton X-100, TW 20: Tween 20)

The esterolytic activity of PsEst_yfh_ in the presence of various metal ions (1 and 10 mM) is presented in Fig. 7a. The results show distinct variations in enzyme activity depending on the ion type and concentration. The relative activity of the enzyme in the presence of 1 mM Ni²⁺ and Cu²⁺ increased to 126.74 ± 1.25% and 124.20 ± 2.92%, respectively (*P < 0.001*). In contrast, a significant loss (>60%) in the enzyme activity was observed with increasing the concentration of these ions to 10 mM (*P < 0.001*). The regulatory effect of Cu²⁺ and Ni²⁺ ions at low concentrations on enzyme activity may be due to their ability to stabilize enzyme conformation and facilitate substrate binding by forming coordinative bonds with the side chains of amino acids such as histidine, aspartate, and glutamate (Kim et al. 2008). On the other hand, these ions in excessive concentrations may have inhibited the enzyme by conformational changes that block the catalytic site, resulting from binding of ions to the imidazole rings of histidines and thiol groups of cysteines, or by generating the reactive oxygen species that can attack the amino acids in the active site due to their strong Lewis acid character (Macomber and Imlay 2009; Halliwell and Gutteridge 2015). It has been determined that Ca²⁺ ions at of 1 mM and 10 mM concentrations enhanced the esterolytic activity of PsEst_yfh_, which represents a significant advantage for biotechnological applications where calcium salts are widely used (*P < 0.001* for 1 mM, *P < 0.01* for 10 mM). Ca²⁺ is a known stimulator and cofactor for many hydrolase enzymes and contributes to the optimal conformation of the catalytic triad (Ser-His-Asp) by establishing electrostatic interactions with negatively charged amino acid residues close to the active site, especially in esterases and lipases (Jaeger and Eggert 2002; Singh et al. 2024). The relative enzyme activity in the presence of Co^2+^, K^+^, Mg^2+^, and Fe^2+^ at 1 mM concentration in the reaction mixture was 79.60 ± 4.69%, 75.10 ± 1.71%, 72.89 ± 1.35%, and 71.73 ± 0.75%, respectively (*P < 0.001*). Elevated concentrations of Mg^2+^ resulted in a limited inhibitory effect (*P < 0.001*), whereas Fe^2+^, Co^2+^, and K^+^ diminished the enzyme activity by ≥ 50% at higher concentrations (*P < 0.001*).

The effect of various chemicals at 1 and 10 mM concentrations on the activity of PsEst_yfh_ is shown in Fig. 7b. It was obvious that chemicals influenced the enzyme in a concentration-dependent manner. In the presence of 1 mM βME, there was a slight decrease (~ 25%) in the enzyme activity (*P < 0.001*), whilst no activity was detected as the concentration of βME was raised to 10 mM. The inhibitory effect of βME does not seem to be related to the reduction of the disulfide bond since it is not in the enzyme structure. Nevertheless, when the concentration of βME was increased, it may have sterically or chemically hindered enzyme-substrate interactions by reacting with the sulfhydryl group of cysteine (Cys)-161 (Sondhi et al. 2021). Addition of SDS at concentrations of 1 or 10 mM to the reaction mixture led to significant enzyme inactivation (*P < 0.001*), probably because SDS causes conformational changes in the enzyme structure by disrupting critical hydrophobic interactions in the active site (Hou et al. 2020). Similarly, the presence of EDTA led to a marked decrease in enzyme activity, with relative activities of 46.44 ± 3.15% (*P < 0.001*) and 28.75 ± 1.11% (*P < 0.001*) at its 1 mM and 10 mM concentrations, respectively. A non-ionic detergent, Triton X-100, did not affect the activity at low concentrations (*P > 0.05*), but it significantly reduced the activity at high concentrations (*P < 0.001*), similar to esterases from *H. salinarum* NCR-1 (Ortega-de la Rosa et al., 2024), *E. antarcticum* (Hwang et al., 2023), and *Streptomyces lividans* TK24 (Fang et al., 2024). On the other hand, in the presence of another non-ionic detergent, Tween 20, at a concentration of 1 mM, the decrease in activity (~ 22.57%) was statistically significant (*P < 0.001*).

**Fig. 8:**
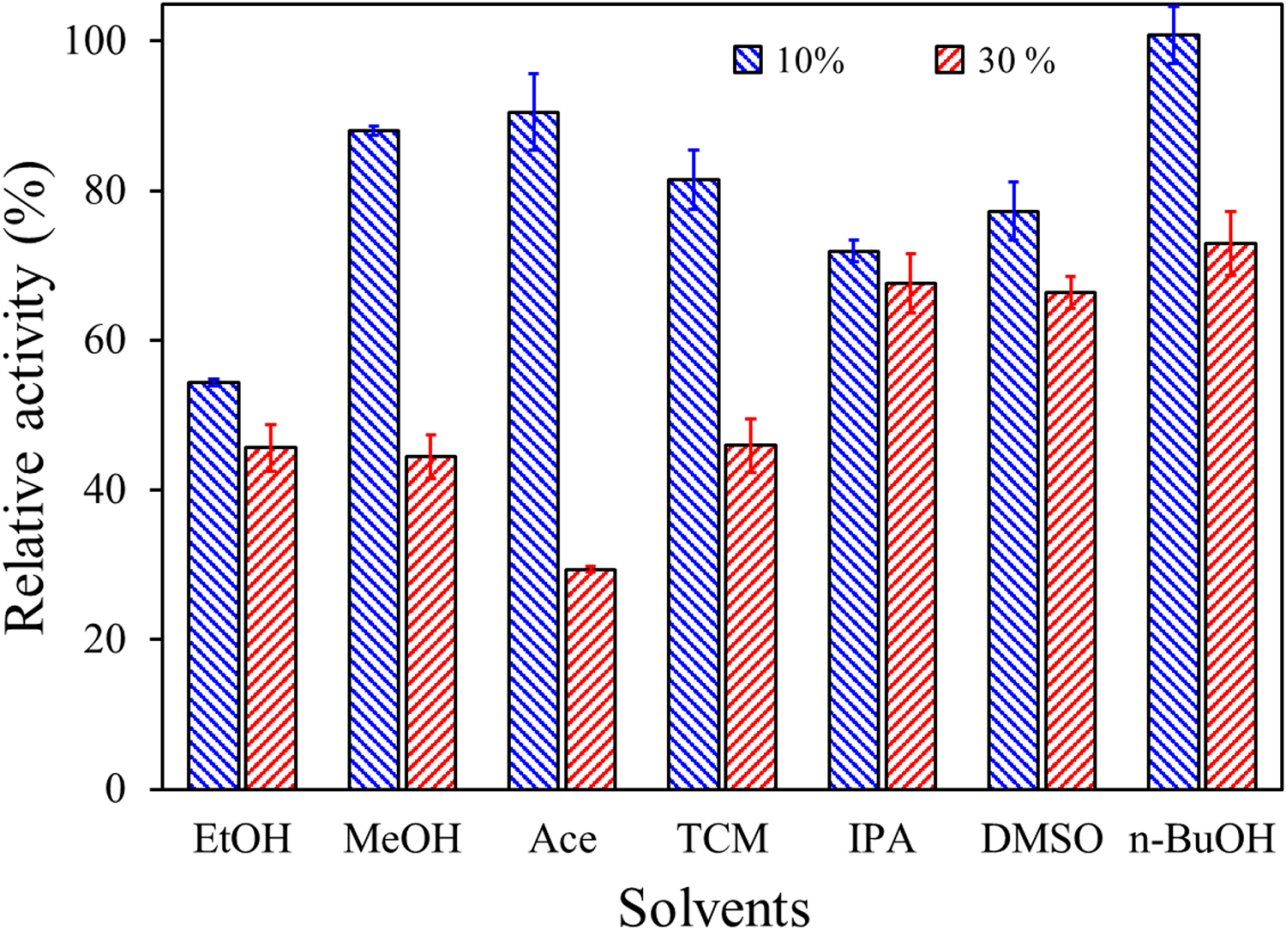
Effect of solvents (Ethanol: EtOH, Methanol: MeOH, Acetone: Ace, Chloroform: TCM, Isopropanol: IPA, Dimethyl sulfoxide: DMSO, Butanol: n-BuOH) on PsEst_yfh_ activity. Assays were carried out under optimal conditions, incorporating each solvent at 10% (v/v), marked by blue diagonal lines, or 30% (v/v), marked by red diagonal lines. Enzyme activity without solvent served as 100%. Data from three replicate experiments was illustrated as the mean with standard error bars

The influence of solvents, namely ethanol, methanol, acetone, chloroform, isopropanol, DMSO, and butanol, on enzyme activity is illustrated in Fig. 8. Accordingly, the enzyme fully retained its activity in the presence of 10% (v/v) butanol, a moderately hydrophobic solvent with a relatively long carbon chain (*P > 0.05*). In comparison, its relative activity was more than 70% when the butanol concentration was increased to 30% (*P < 0.001*). PsEst_yfh_ was slightly inhibited by 10% and 30% concentrations of isopropanol and DMSO (*P < 0.001*). When methanol and acetone were added to the reaction mixture at a final concentration of 10%, a relatively small decrease in the catalytic activity of the enzyme was observed (*P > 0.05*), but increasing the concentration of the solvents in the reaction mixture to 30% resulted in a loss of about 55% and 70% of the enzyme activity, respectively (*P < 0.001*). Furthermore, ethanol in the reaction mixture significantly diminished the enzyme activity at 10% and 30% concentrations. The findings indicate that PsEst_yfh_ could be used in biotechnological applications involving organic solvents, especially with moderate hydrophobicity.

**Fig. 9:**
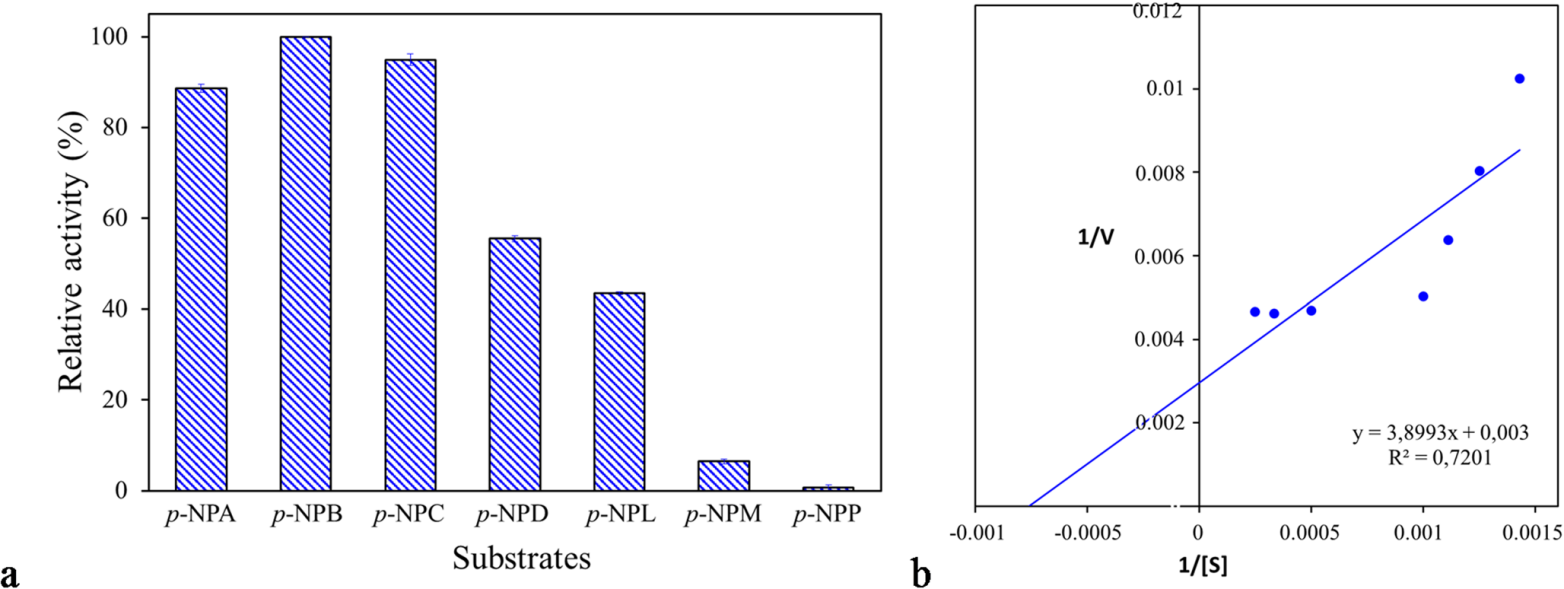
**a** Substrate specificity of PsEst_yfh_ (*p*-NPA: *p*-nitrophenyl acetate, *p*-NPB: *p*-nitrophenyl butyrate, *p*-NPC: *p*-nitrophenyl caprylate, *p*-NPD: *p*-nitrophenyl decanoate, *p*-NPL: *p*-nitrophenyl laurate, *p*-NPM: *p*-nitrophenyl myristate, and *p*-NPP: *p*-nitrophenyl palmitate). The experiments were conducted in a 50 mM Tris-HCl buffer (pH 9) at 20 °C. In calculating the relative activities, the observed maximum activity was assumed to be 100%. **b** Lineweaver-Burk graph plotted by using the data of the esterase assays in the presence of *p*-NPB (0–4 mM) as a substrate

The evaluation of PsEst_yfh_’s ability to hydrolyze *p*-NP esters with different fatty acid chains (C_2_-C_16_) at pH 9 and 20 °C revealed that it preferred short-chain esters as substrates (Fig. 9a). PsEst_yfh_ exhibited the highest catalytic efficiency against *p*-NPB, followed by *p*-NPC (94.94 ± 2.28%), p-NPA (88.56 ± 1.52%), and *p*-NPD (55.49 ± 1.13%). The relative activity of the enzyme in reactions carried out in the presence of *p*-NPL as a substrate was recorded as 43.48 ± 0.45%. On the other hand, the enzyme did not exhibit significant catalytic activity against the *p*-NPM substrate (6.44 ± 0.87%), and its activity toward *p*-NPP was so weak that it was nonexistent. The differences in the mean values of PsEst_yfh_’s hydrolytic activity against the tested substrates were greater than expected by chance (*P < 0.01*). Based on the above findings, it appears that PsEst_yfh_ exhibits functional properties similar to a typical esterase like Tan410 (Yao et al. 2021), LIP2 (Mohanan et al. 2022), E53 (Ding et al. 2022), Gsu768 (Zhang et al. 2024), Dhs82 (Wang et al. 2025b), Epux1 (Soto-Hernández et al. 2025), and Est4L (Park et al. 2021); however, it shows a relatively broader substrate specificity compared to esterases from *D. radiodurans* (Zhang et al., 2021), *Klebsiella aerogenes* (Gao et al., 2021), *Alicycliphilus denitrificans* BQ1 (Fuentes-Jaime et al., 2022), *Lysinibacillus* sp. (Matrawy et al., 2022), *Staphylococcus chromogenes* (Hwang et al., 2022), and *Microbacterium chocolatum* SIT101 (Li et al., 2024).

Kinetic analysis performed with *p*-NPB revealed that PsEst_yfh_ exhibited behavior consistent with classical Michael-Menten kinetics (Fig. 9b). Furthermore, the *K*_*m*_ and *V*_*max*_ values of the enzyme were calculated from the Lineweaver-Burk plot as 129.97 µM and 33.3 µmol/min, respectively. The results show that PsEstyfh’s affinity towards *p*-NPB is higher than that of its many counterparts (Barman and Dkhar 2022; Santos et al. 2023).

### Molecular docking

**Fig. 10:**
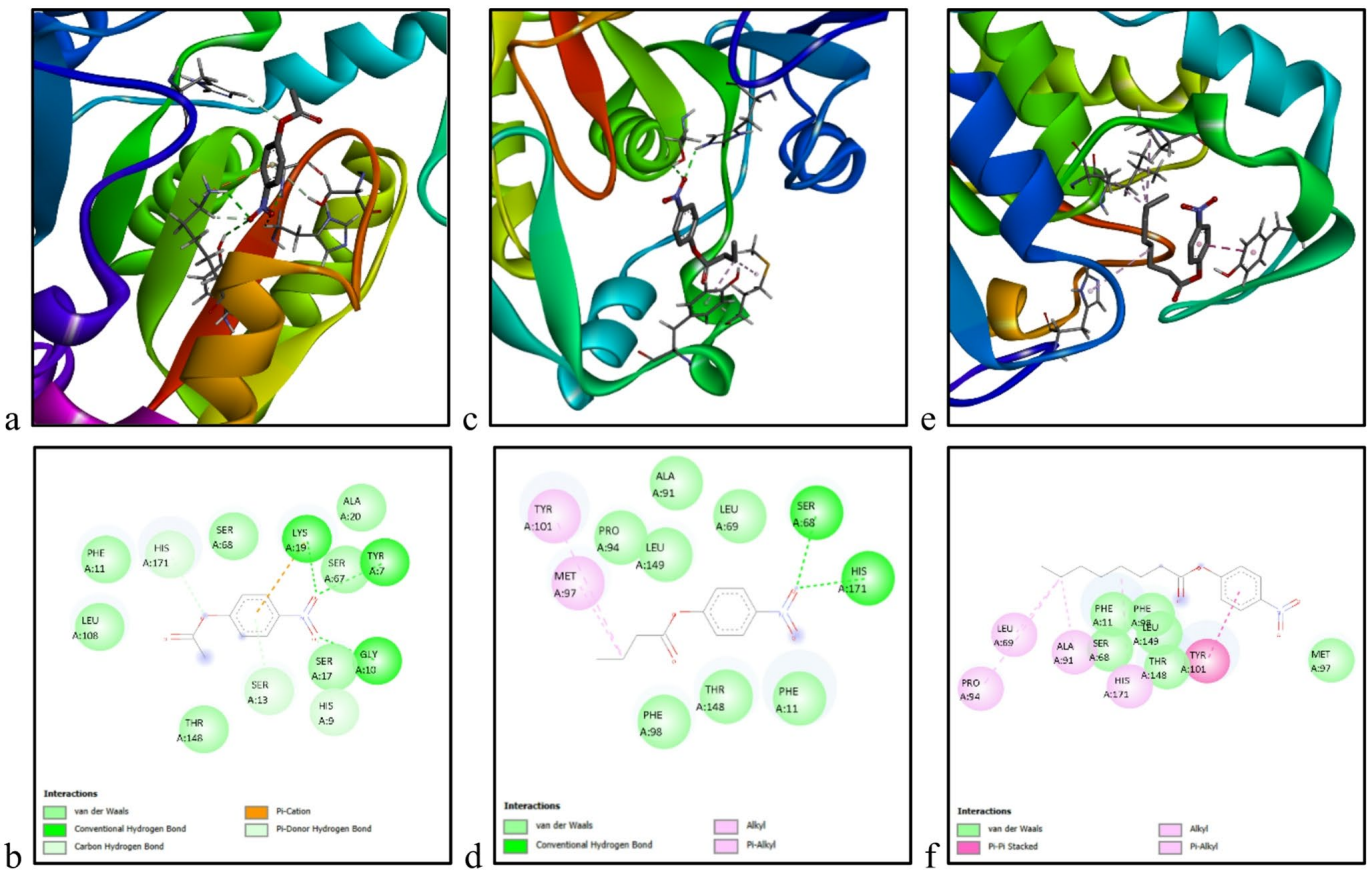
3D representations of the individual interactions between PsEst_yfh_ and *p*-NPA (**a**), p-NPB (**c**), and *p*-NPC (**e**). 2D representations of the individual interactions between PsEst_yfh_ and *p*-NPA (**b**), *p*-NPB (**d**), and *p*-NPC (**f**). Interactions between the enzyme and substrates were investigated using molecular docking with AutoDock Vina v1.1.2, based on three-dimensional structures generated with Swiss-Model and the PubChem database. UCSF Chimera and Discovery Studio Visualizer were used during the analysis. For each substrate, only the best-scoring enzyme–substrate complexes were presented (*p*-NPA: *p*-nitrophenyl acetate, *p*-NPB: *p*-nitrophenyl butyrate, and *p*-NPC: *p*-nitrophenyl caprylate)

Substrates, including *p*-NPA, *p*-NPB, and *p*-NPC, which exhibited high catalytic preference in functional assays, were docked against PsEst_yfh_. The models, built based on the interaction with the lowest binding energy (Vina score), are shown in Fig. 10. Calculated binding energies for *p*-NPA, *p*-NPB, and *p*-NPC were − 5.4 kJ/mol, -6.0 kJ/mol, and − 5.8 kJ/mol, respectively. In this context, the substrate binding affinity order (p-NPA > p-NPB > p-NPH) was found to be consistent with functional analyses. Three conventional hydrogen bonds (TYR 7, GLY 10, and LYS 19), two π-donor hydrogen bonds (HIS 9, SER 13, and HIS 171), seven van der Waals interactions (SER 17, PHE 11, ALA 20, SER 67, SER 68, LEU108, and THR 148), and a π-cation bond (LYS 19) were observed between the enzyme and *p*-NPA. The model constructed with *p*-NPB indicated that the SER 68 and HIS 171 residues of PsEst_yfh_ interact with the substrate via conventional hydrogen bonds. It confirms the role of Ser 68 in the conserved Gly-X-Ser-X-Gly motif of esterases as an active site residue (Sarkar et al. 2020). It revealed that the substrate interacts with the enzyme’s MET 97 and TYR 101 residues via alkyl and π-alkyl bonds, respectively. In addition, seven van der Waals interactions (PHE 11, LEU 69, ALA 91, PRO 94, PHE 98, THR 148, and LEU 149) were detected between PsEst_yfh_ and *p*-NPB. According to the model shown in Fig. 10e and f, despite no hydrogen bond being formed between *p*-NPC and the amino acid residues of the enzyme, a π-π stacked bond (TYR 101), a π-alkyl bond (HIS 171), three alkyl bonds (LEU 69, ALA 91, and PRO 94), and six van der Waals interactions (PHE 11, SER 68, MET 97, PHE 98, THR 148, and LEU 149) were detected between the PsEst_yfh_ and *p*-NPC.

### PsEst_yfh_-mediated reduction of antimicrobial activity of azithromycin

**Fig. 11:**
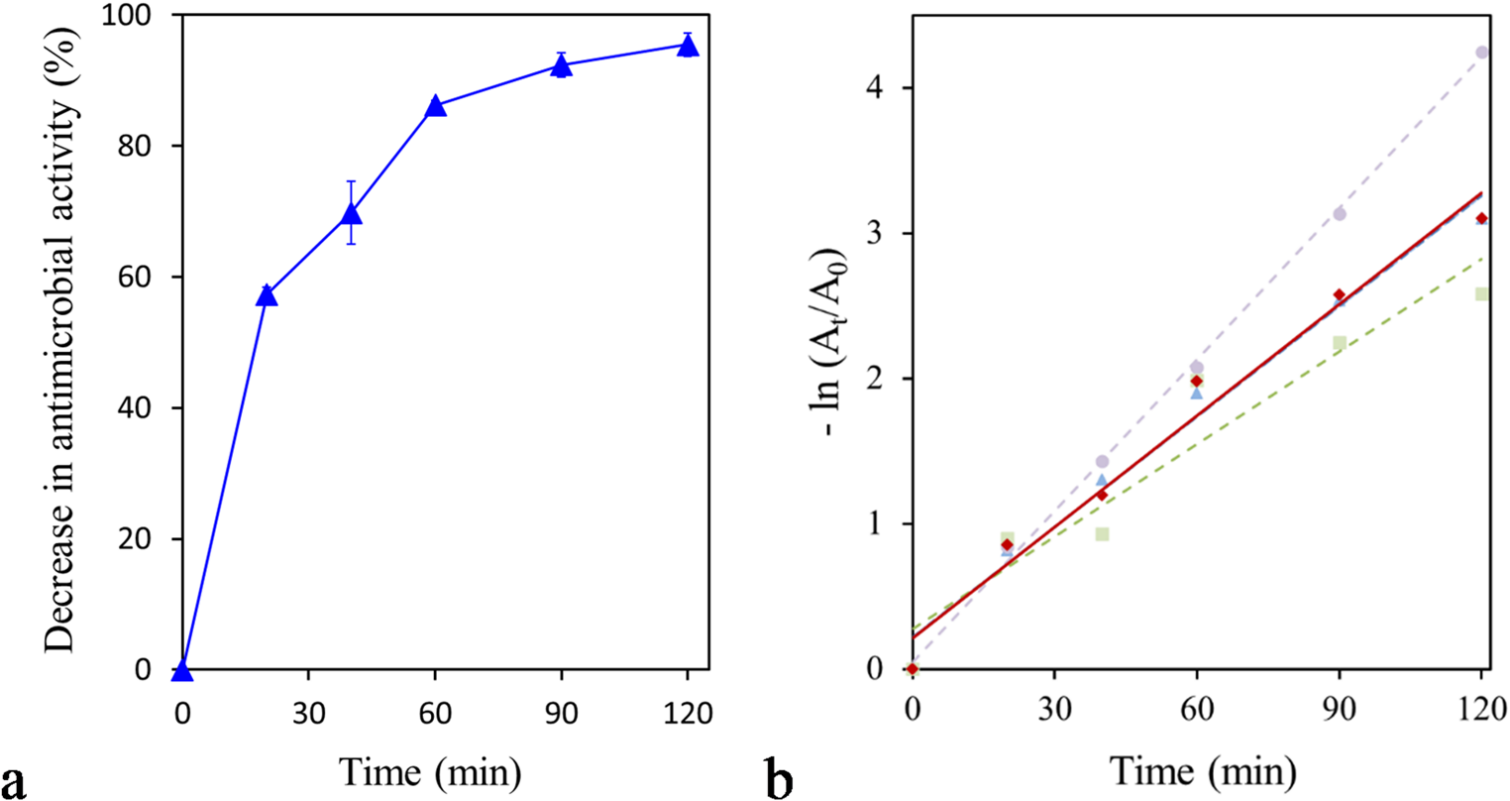
**a** Time-dependent decrease in the antimicrobial activity of azithromycin in the presence of PsEst_yfh_. **b** Kinetic analysis of the decrease in the antimicrobial activity of azithromycin mediated by PsEst_yfh_ under the assumption of pseudo-first-order kinetics. The red solid line represents the average of the three individual kinetic profiles shown by the dashed lines

The time-dependent effect of PsEst_yfh_ on the antimicrobial activity of azithromycin was evaluated. As shown in Fig. 11a, a rapid decrease in azithromycin’s antimicrobial activity was detected in the early stages of the reaction. Within the first 20 min, a decrease of approximately 57.35 ± 1.87% in activity was observed, and this decrease rose to 86.25 ± 1.22% by the 60th min. Extending the reaction time resulted in a maximum decrease of approximately 95.50 ± 3.08% after 120 min, indicating that azithromycin is highly susceptible to PsEst_yfh_. The sharp initial decline likely implies a rapid enzymatic interaction resulting from the presence of ester-linked functional groups that are readily accessible to esterase activity. PsEstyfh decreases the antimicrobial activity of the azithromycin by hydrolyzing the ester bond within the macrolactone ring through a nucleophilic attack initiated by the catalytic serine residue. This enzymatic hydrolysis results in the opening of the macrocyclic ring, subsequently leading to the loss of its ribosomal binding affinity (Zieliński et al. 2021; Kelly et al. 2026). The surface-accessible active site architecture, facilitated by the enzyme’s small molecular size, enables the rapid hydrolysis of azithromycin substrates without significant steric hindrance. To the best of our knowledge, no study in the literature has evaluated the use of esterases to reduce azithromycin’s antimicrobial activity under similar conditions and methodologies. However, the reported effects of previously characterized esterases on other macrolide antibiotics underscore the comparative superiority of PsEst_yfh_ over its counterparts. The esterase OCA_1 was reported to remove tylosin completely within 30 min, tilmicosin within 2 h, and tildipirosin within 4 h (Tao et al. 2026). In another study, *Enho*-Ere (0.1 mg/mL) was found to convert 100% of erythromycin and clarithromycin, and 50% of roxithromycin at a concentration of 0.8 g/L over a 12-hour reaction period (Zhang et al. 2023).

Kinetic analysis (Fig. 11b) revealed that the decrease in the antimicrobial activity of azithromycin followed pseudo-first-order kinetics during the early reaction phase (0–60 min), as indicated by the increase in − ln(Aₜ/A₀) from 0 to 1.98. The substantially smaller increment observed at later time points, with − ln(Aₜ/A₀) reaching 3.10 at 120 min, indicates saturation of the enzymatic process. The high catalytic performance of PsEst_yfh_ at low temperatures and under alkaline conditions is a distinctive feature that sets it apart from many related enzymes concerning its applicability for macrolide antibiotic removal. For instance, the cold-active erythromycin esterase (EstB) from *Alcanivorax dieselolei* B-5(T) exhibits maximum catalytic activity at 20 °C; however, its efficiency declines significantly at pH values above 8.5 (Zhang et al. 2014). Furthermore, research on *Enho*-Ere derived from *Enterobacter hormaechei* indicates that its high catalytic activity is confined to a pH range of 6.0–8.0 and temperatures between 40 °C and 55 °C (Zhang et al. 2023). Similarly, EreA from *Rhodococcus gordonii* rjjtx-2 has been reported to lack significant activity under both alkaline conditions and at low temperatures (Ren et al. 2022). Furthermore, the small molecular size of PsEst_yfh_ represents a critical strategic advantage for protein engineering. Unlike many other esterases with macrolide removal activity, such as erythromycin esterase from *Pseudomonas* sp. GD100 (Kim et al., 2002), EstB (Zhang et al., 2014), EreA (Ren vd., 2022), and *Enho*-Ere (Zhang et al., 2023), the compact structure of PsEst_yfh_ may facilitate its integration into multifunctional fusion enzyme systems, ensuring better stability and secretion efficiency in heterologous hosts (Elleuche 2015). Overall, these results demonstrate that PsEst_yfh_ effectively reduces the antimicrobial activity of azithromycin in a time-dependent manner, offering a promising, energy-efficient, and structurally versatile tool for environmental remediation of antibiotic residues in cold and alkaline environments.

### Ecotoxicological assessment

**Fig. 12:**
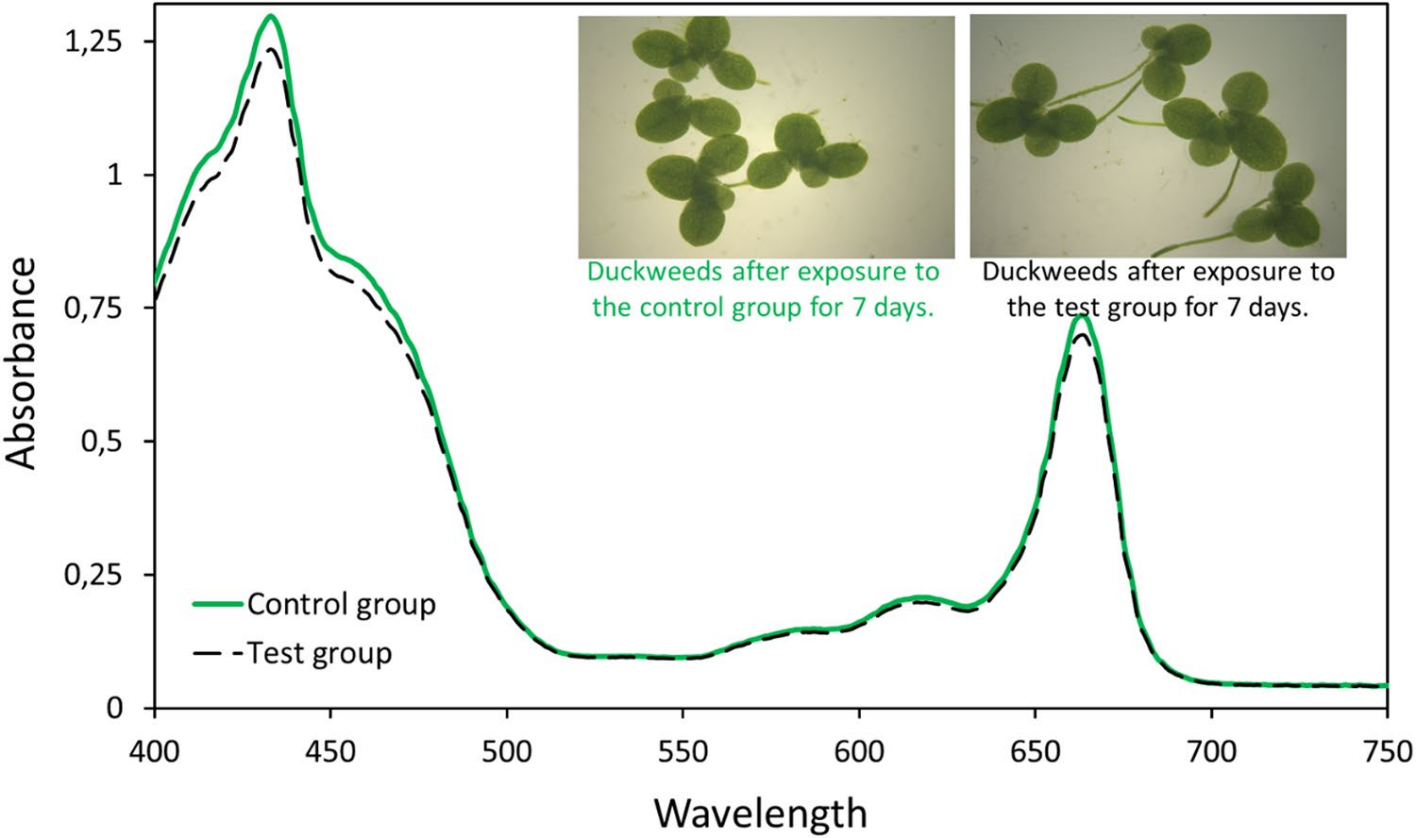
Absorption spectra of duckweed homogenates after exposure to the control group (green straight line) and the test group (black dashed line). The control group was 50 mM Tris-HCl buffer (pH 9), while the test group was the ultrafiltrate of the reaction mixture after azithromycin removal experiments. The control group (green solid line) refers to duckweed grown in a 50 mM Tris-HCl buffer (pH 9), while the test group (black dashed line) represents duckweed grown in the ultrafiltrate of the reaction mixture obtained following an azithromycin removal experiment conducted with PsEstyfh

Ecotoxicological assessment of end-products in the reaction mixture, where the azithromycin antimicrobial activity was reduced (by enzymatic treatment), was first performed based on calculated Percent Inhibition of Growth Rate ($\:\mathrm{\%}{I}_{r}$). The measured $\:\mathrm{\%}{I}_{r}\:$ value at the end of the 7-day experiments indicated minimal inhibition (4.76%) in the test group duckweed, which was not biologically significant compared to the control group (*P > 0.05*). Photosynthetic pigment content is another crucial growth indicator for evaluating toxicity in duckweed. Therefore, the absorption spectra of homogenized duckweed were recorded (Fig. 12). In the current study, it was observed that the photosynthetic pigment content of *L. minor* was not significantly affected from the test group when compared to the control group (*P > 0.05*). The decrease in chlorophyll a, chlorophyll b, and total chlorophyll content was 4.88 ± 0.18%, 4.61 ± 0.50%, and 4.81 ± 0.13%, respectively. To further support the toxicity assessment, the carotenoid contents of the test and control groups were also compared, as carotenoids act as antioxidants that protect chlorophyll pigments and scavenge free radicals, thereby reducing oxidative damage to cellular membranes and DNA. The total carotenoid content in the test group was 3.12 ± 0.21% lower than in the control group (*P = 0.432*).

## Conclusion

The present study reports a previously uncharacterized esterolitic enzyme from *P. sivasensis* R11S16, which was successfully expressed in *E. coli*. The enzyme exhibits a rare combination of desirable biochemical features, including strong activity at cold temperatures, an alkaline pH preference, considerable stability across a broad pH range, moderate tolerance to various additives (NaCl, solvents, metal ions, and chemicals), and a compact molecular structure. These properties make it useful, especially for wastewater treatment applications. Furthermore, the enzyme’s ability to almost eliminate the antimicrobial activity of azithromycin, and the fact that the transformation products released during the process did not cause toxic effects, highlight that PsEst_yfh_ is a promising tool for the biocatalytic removal of macrolide antibiotics and the reduction of the environmental spread of antimicrobial resistance. Beyond environmental applications, the enzyme is also suitable for detergent, textile, and pharmaceutical industries. Additionally, the enzyme’s small molecular size presents a significant advantage for synthetic biology applications, especially in the design of multi-functional fusion enzymes aimed at expanding catalytic versatility for the removal of structurally diverse xenobiotics.

## Supplementary Information

Below is the link to the electronic supplementary material.

Supplementary Material 1

## Data Availability

All data are available from the corresponding author on request. In addition, 16 S rRNA partial sequence of isolate R11S16 is deposited in the GenBank database of NCBI with accession number PP213417 (https://www.ncbi.nlm.nih.gov/nuccore/PP213417). The protein sequences and structural models for PsEst_yfh_ are included within the article and its supplementary materials.

## References

[CR1] Addis TZ, Adu JT, Kumarasamy M, Demlie M (2024) Occurrence of trace-level antibiotics in the msunduzi river: an investigation into South African environmental pollution. Antibiotics 13(2):174. 10.3390/antibiotics13020174 38391560 10.3390/antibiotics13020174 PMC10886320

[CR2] Adıgüzel AO (2020) Production and characterization of thermo-, halo-and solvent-stable esterase from *Bacillus mojavensis* TH309. Biocatal Biotransform 38(3):210–226. 10.1080/10242422.2020.1715370

[CR3] Adigüzel AO, Tunçer M (2016) Production, characterization and application of a xylanase from* Streptomyces* sp. AOA40 in fruit juice and bakery industries. Food Biotechnol 30(3):189–218. 10.1080/08905436.2016.1199383

[CR4] Adigüzel AO, Könen-Adigüzel S, Cilmeli S, Mazmancı B, Yabalak E, Üstün-Odabaşı S, Kaya NG, Mazmancı MA (2023) Heterologous expression, purification, and characterization of thermo-and alkali-tolerant laccase-like multicopper oxidase from *Bacillus mojavensis* TH309 and determination of its antibiotic removal potential. Arch Microbiol 205(8):287. 10.1007/s00203-023-03626-5 37454356 10.1007/s00203-023-03626-5

[CR5] Adıgüzel AO, Şen F, Könen-Adıgüzel S, Kıdeyş AE, Karahan A, Doruk T, Tunçer M (2024) Identification of cutinolytic esterase from microplastic-associated microbiota using functional metagenomics and its plastic degrading potential. Mol Biotechnol 66(10):2995–3012. 10.1007/s12033-023-00916-7 37815749 10.1007/s12033-023-00916-7

[CR6] Adıgüzel SK, Odabaşı SÜ, Yabalak E, Kaya NG, Mazmancı B, Adıgüzel AO (2025) A durable biocatalyst constructed by immobilization of recombinant laccase-like multicopper oxidase from Bacillus mojavensis onto hazelnut shell hydrochar: Its characterization and potential for use in the degradation of sulfamethoxazole and diclofenac. Int J Biol Macromol. 10.1016/j.ijbiomac.2025.144640 10.1016/j.ijbiomac.2025.144640 40436172

[CR7] Aranda J, Cerqueira NMFSA, Fernandes PA, Roca M, Tuñón I, Ramos MJ (2014) The catalytic mechanism of carboxylesterases: a computational study. Biochemistry 53(36):5820–5829. 10.1021/bi500934j 25101647 10.1021/bi500934j

[CR8] Bapat MS, Singh H, Shukla SK, Singh PP, Vo DVN, Yadav A, Goyal A, Sharma A, Kumar D (2022) Evaluating green silver nanoparticles as prospective biopesticides: an environmental standpoint. Chemosphere 286:131761. 10.1016/j.chemosphere.2021.131761 34375828 10.1016/j.chemosphere.2021.131761

[CR9] Barman D, Dkhar MS (2022) Characterization and purification of esterase from *Cellulomonas fimi* DB19 isolated from* Zanthoxylum armatum* with its possible role in diesel biodegradation. Arch Microbiol 204(9):580. 10.1007/s00203-022-03210-3 36030426 10.1007/s00203-022-03210-3

[CR10] Barzkar N, Sohail M, Tamadoni Jahromi S, Gozari M, Poormozaffar S, Nahavandi R, Hafezieh M (2021) Marine bacterial esterases: emerging biocatalysts for industrial applications. Appl Biochem Biotechnol 193(4):1187–1214. 10.1007/s12010-020-03483-8 33411134 10.1007/s12010-020-03483-8

[CR11] Basiglini E, Pintore M, Forni C (2018) Effects of treated industrial wastewaters and temperatures on growth and enzymatic activities of duckweed (*Lemna minor* L). Ecotoxicol Environ Saf 153:54–59. 10.1016/j.ecoenv.2018.01.053 29407738 10.1016/j.ecoenv.2018.01.053

[CR12] Boyko KM, Kryukova MV, Petrovskaya LE, Kryukova EA, Nikolaeva AY, Korzhenevsky DA, Lomakina GY, Novototskaya-Vlasova KA, Rivkina EM, Dolgikh DA, Kirpichnikov MP, Popov VO (2021) Structural and biochemical characterization of a cold-active PMGL3 esterase with unusual oligomeric structure. Biomolecules 11(1):57. 10.3390/biom11010057 33466452 10.3390/biom11010057 PMC7824956

[CR13] Bradford MM (1976) A rapid and sensitive method for the quantitation of microgram quantities of protein utilizing the principle of protein-dye binding. Anal Biochem 72(1–2):248–254. 10.1016/0003-2697(76)90527-3 942051 10.1016/0003-2697(76)90527-3

[CR14] Brault G, Shareck F, Hurtubise Y, Lépine F, Doucet N (2012) Isolation and characterization of EstC, a new cold-active esterase from* Streptomyces coelicolor* A3 (2). PLoS ONE 7(3):e32041. 10.1371/journal.pone.0032041 22396747 10.1371/journal.pone.0032041 PMC3292560

[CR15] Browne AJ, Chipeta MG, Haines-Woodhouse G, Kumaran EP, Hamadani BHK, Zaraa S, Henry NJ, Deshpande A, Reiner RC, Day NPJ, Lopez AD, Dunachie S, Moore CE, Stergachis A, Hay SI, Dolecek C (2021) Global antibiotic consumption and usage in humans, 2000–18: a spatial modelling study. Lancet Planet Health 5(12):e893–e904. 10.1016/S2542-5196(21)00280-1 34774223 10.1016/S2542-5196(21)00280-1 PMC8654683

[CR16] Buchholz-Cleven BE, Rattunde B, Straub KL (1997) Screening for genetic diversity of isolates of anaerobic Fe (II)-oxidizing bacteria using DGGE and whole-cell hybridization. Syst Appl Microbiol 20(2):301–309. 10.1016/S0723-2020(97)80077-X

[CR17] Cao L, Xue D, Liu X, Wang C, Fang D, Zhang J, Gong C (2023) Ferulic acid production from wheat bran by integration of enzymatic pretreatment and a cold-adapted carboxylesterase catalysis. Bioresour Technol 385:129435. 10.1016/j.biortech.2023.129435 37399964 10.1016/j.biortech.2023.129435

[CR18] Cedeño-Muñoz JS, Aransiola SA, Reddy KV, Ranjit P, Victor-Ekwebelem MO, Oyedele OJ, Pérez-Almeida IB, Maddela NR, Rodríguez-Díaz JM (2024) Antibiotic resistant bacteria and antibiotic resistance genes as contaminants of emerging concern: occurrences, impacts, mitigations and future guidelines. Sci Total Environ 952:175906. 10.1016/j.scitotenv.2024.175906 39226958 10.1016/j.scitotenv.2024.175906

[CR19] Chalita M, Kim YO, Park S, Oh HS, Cho JH, Moon J, Baek N, Moon C, Lee K, Yang J, Nam GG, Jung Y, Na SI, Bailey MJ, Chun J (2024) EzBioCloud: a genome-driven database and platform for microbiome identification and discovery. Int J Syst Evol MicroBiol 74(6):006421. 10.1099/ijsem.0.006421 38888585 10.1099/ijsem.0.006421 PMC11261700

[CR20] Choudhary M, Laurie CC (1991) Use of in vitro mutagenesis to analyze the molecular basis of the difference in Adh expression associated with the allozyme polymorphism in *Drosophila melanogaster*. Genetics 129(2):481–488. 10.1093/genetics/129.2.481 1743488 10.1093/genetics/129.2.481 PMC1204637

[CR21] Chun J, Goodfellow M (1995) A phylogenetic analysis of the genus Nocardia with 16S rRNA gene sequences. Int J Syst Evol MicroBiol 45(2):240–245. 10.1099/00207713-45-2-240 10.1099/00207713-45-2-240 7537058

[CR22] Chung CT, Miller RH (1993) Preparation and storage of competent *Escherichia coli* cells. In: Methods in enzymology, Vol 218, pp 621–627. Academic Press. 10.1016/0076-6879(93)18045-E 10.1016/0076-6879(93)18045-e 8510550

[CR23] Cilmeli S, Doruk T, Könen-Adıgüzel S, Adıgüzel AO (2024) A thermostable and acidophilic mannanase from Bacillus mojavensis: its sustainable production using spent coffee grounds, characterization, and application in grape juice processing. Biomass Convers Biorefinery 14(3):3811–3825. 10.1007/s13399-022-02602-1

[CR24] de Boer S, Sastre D, Castillo A, Méndez SB, Hollmann F, Lores M, Schäffer A, Moreira MT (2025) Advancing the enzymatic removal of antibiotics with unspecific peroxygenase and vanadium chloroperoxidase. J Environ Chem Eng 13(2):115795. 10.1016/j.jece.2025.115795

[CR25] De Santi C, Leiros HKS, Di Scala A, de Pascale D, Altermark B, Willassen NP (2016) Biochemical characterization and structural analysis of a new cold-active and salt-tolerant esterase from the marine bacterium *Thalassospira* sp. Extremophiles 20:323–336. 10.1007/s00792-016-0824-z 27016194 10.1007/s00792-016-0824-z

[CR26] Dhaulaniya AS, Balan B, Kumar M, Agrawal PK, Singh DK (2019) Cold survival strategies for bacteria, recent advancement and potential industrial applications. Arch Microbiol 201(1):1–16. 10.1007/s00203-018-1602-3 30478730 10.1007/s00203-018-1602-3

[CR27] Dhindwal P, Myziuk I, Ruzzini A (2023) Macrolide esterases: current threats and opportunities. Trends Microbiol 31(12):1199–1201. 10.1016/j.tim.2023.08.008 37689489 10.1016/j.tim.2023.08.008

[CR28] Ding Y, Nie L, Yang XC, Li Y, Huo YY, Li Z, Gao Y, Cui HL, Li J, Xu XW (2022) Mechanism and structural insights into a novel esterase, E53, isolated from *Erythrobacter longus*. Front Microbiol 12:798194. 10.3389/fmicb.2021.798194 35069500 10.3389/fmicb.2021.798194 PMC8767022

[CR30] Dong J, Zhao W, Gasmalla MA, Sun J, Hua X, Zhang W, Han L, Fan Y, Feng Y, Shen Q, Yang R (2015) A novel extracellular cold-active esterase of* Pseudomonas* sp. TB11 from glacier 1: differential induction, purification and characterisation. J Mol Catal B Enzymat 121:53–63. 10.1016/j.molcatb.2015.07.015

[CR29] Dong J, Gasmalla MA, Zhao W, Sun J, Liu W, Wang M, Han L, Yang R (2017) Characterization of a cold-adapted esterase and mutants from a psychotolerant *Pseudomonas* sp. strain. Biotechnol Appl Chem 64(5):686–699. 10.1002/bab.1525 10.1002/bab.1525 27405585

[CR31] Duman M, Mulet M, Saticioglu IB, Altun S, Gomila M, Lalucat J, Garcia-Valdes E (2020) *Pseudomonas sivasensis* sp. nov. isolated from farm fisheries in Turkey. Syst Appl Microbiol 43(4):126103. 10.1016/j.syapm.2020.126103 32690194 10.1016/j.syapm.2020.126103

[CR32] Eke SM, Cua A (2025) Invisible engines of resistance: how global inequities drive antimicrobial failure. Antibiotics 14(7):659. 10.3390/antibiotics14070659 40723962 10.3390/antibiotics14070659 PMC12291774

[CR33] Elleuche S (2015) Bringing functions together with fusion enzymes-from nature’s inventions to biotechnological applications. Appl Microbiol Biotechnol 99(4):1545–1556. 10.1007/s00253-014-6315-1 25535094 10.1007/s00253-014-6315-1

[CR34] Esteban-Torres M, Mancheño JM, De Las Rivas B, Muñoz R (2014) Characterization of a cold-active esterase from* Lactobacillus plantarum* suitable for food fermentations. J Agric Food Chem 62(22):5126–5132. 10.1021/jf501493z 24856291 10.1021/jf501493z

[CR35] Ezra R, Vanti G, Masaphy S (2025) Sustainable, targeted, and cost-effective laccase-based bioremediation technologies for antibiotic residues in the ecosystem: a comprehensive review. Biomolecules 15(8):1138. 10.3390/biom15081138 40867583 10.3390/biom15081138 PMC12383733

[CR36] Fang J, An L, Yu J, Ma J, Zhou R, Wang B (2024) Characterization of a novel carboxylesterase from *Streptomyces lividans* TK24 and site-directed mutagenesis for its thermostability. J Biosci Bioeng 138(3):181–187. 10.1016/j.jbiosc.2024.05.001 38871580 10.1016/j.jbiosc.2024.05.001

[CR37] Fekete-Kertesz I, Kunglne-Nagy Z, Gruiz K, Magyar A, Farkas E, Molnar M (2015) Assessing toxicity of organic aquatic micropollutants based on the total chlorophyll content of *Lemna minor* as a sensitive endpoint. Periodica Polytech Chem Eng 59(4):262–271. 10.3311/PPch.8077

[CR38] Felsenstein J (1985) Confidence limits on phylogenies: an approach using the bootstrap. Evolution 39(4):783–791. 10.1111/j.1558-5646.1985.tb00420.x 28561359 10.1111/j.1558-5646.1985.tb00420.x

[CR39] Fu J, Leiros HKS, de Pascale D, Johnson KA, Blencke HM, Landfald B (2013) Functional and structural studies of a novel cold-adapted esterase from an Arctic intertidal metagenomic library. Appl Microbiol Biotechnol 97:3965–3978. 10.1007/s00253-012-4276-9 22832985 10.1007/s00253-012-4276-9

[CR40] Fuentes-Jaime J, Vargas-Suárez M, Cruz-Gómez MJ, Loza-Tavera H (2022) Concerted action of extracellular and cytoplasmic esterase and urethane-cleaving activities during Impranil biodegradation by *Alicycliphilus denitrificans* BQ1. Biodegradation 33(4):389–406. 10.1007/s10532-022-09989-8 35633408 10.1007/s10532-022-09989-8

[CR41] Gao H, Zhu R, Li Z, Wang W, Liu Z, Hu N (2021) Improving the catalytic efficiency and substrate affinity of a novel esterase from marine Klebsiella aerogenes by random and site-directed mutation. World J Microbiol Biotechnol 37(6):106. 10.1007/s11274-021-03069-4 34037848 10.1007/s11274-021-03069-4

[CR42] Gao Z, Tan J, Khan MF, Chugh G, Schmidt O, Ma L, Bu D (2025) Emerging microbial and enzymatic approaches for sustainable antibiotic biodegradation in livestock manure to mitigate water pollution risks. Water 17(20):2960. 10.3390/w17202960

[CR43] Gasteiger E, Hoogland C, Gattiker A, Duvaud SE, Wilkins MR, Appel RD, Bairoch A (2005) Protein identification and analysis tools on the ExPASy server. The proteomics protocols handbook. Humana, Totowa, NJ, pp 571–607. 10.1385/1-59259-890-0:571

[CR44] Halliwell B, Gutteridge JM (2015) Free radicals in biology and medicine. Oxford University Press

[CR45] Hashim NHF, Mahadi NM, Illias RM, Feroz SR, Abu Bakar FD, Murad AMA (2018) Biochemical and structural characterization of a novel cold-active esterase-like protein from the psychrophilic yeast *Glaciozyma antarctica*. Extremophiles 22:607–616. 10.1007/s00792-018-1021-z 29556723 10.1007/s00792-018-1021-z

[CR46] He J, Zhang Y, Wu L, Wang Y, Zhang H, Liu Z, Shi X (2024) Characterization of a novel esterase belonging to family V from *Marinobacter flavimaris*. J Ocean Univ China 23(1):221–232. 10.1007/s11802-024-5664-3

[CR47] Hou H, He H, Wang Y (2020) Effects of SDS on the activity and conformation of protein tyrosine phosphatase from thermus thermophilus HB27. Sci Rep 10(1):3195. 10.1038/s41598-020-60263-4 32081966 10.1038/s41598-020-60263-4 PMC7035334

[CR48] Hwang J, Jeon S, Lee MJ, Yoo W, Chang J, Kim KK, Lee JH, Do H, Kim D, Kim TD (2022) Identification, characterization, and preliminary X-ray diffraction analysis of a novel esterase (Sc Est) from *Staphylococcus chromogenes*. Crystals 12(4):546. 10.3390/cryst12040546

[CR49] Hwang J, Yoo W, Shin SC, Kim KK, Kim HW, Do H, Lee JH (2023) Structural and biochemical insights into Bis (2-hydroxyethyl) terephthalate degrading carboxylesterase isolated from Psychrotrophic bacterium *Exiguobacterium antarcticum*. Int J Mol Sci 24(15):12022. 10.3390/ijms241512022 37569396 10.3390/ijms241512022 PMC10418727

[CR50] Jaeger KE, Eggert T (2002) Lipases for biotechnology. Curr Opin Biotechnol 13(4):390–397. 10.1016/S0958-1669(02)00341-5 12323363 10.1016/s0958-1669(02)00341-5

[CR51] Jiang H, Zhang S, Gao H, Hu N (2016) Characterization of a cold-active esterase from* Serratia* sp. and improvement of thermostability by directed evolution. BMC Biotechnol 16:1–11. 10.1186/s12896-016-0235-3 26800680 10.1186/s12896-016-0235-3 PMC4722774

[CR52] Jiang W, Zhang S, Xu C, Sun H, Xing Z, Liang X, He H, Li Q (2025) Sustainable biodegradation of antibiotic residues in aqueous environment using OXA-48 β-lactamase encapsulated in spherical mesoporous covalent organic frameworks. J Hazard Mater. 10.1016/j.jhazmat.2025.138856 10.1016/j.jhazmat.2025.138856 40499418

[CR53] Karakaya E (2025) Kaçkar Dağları’ndan Psikrofilik ve Psikrotolerant Bakterilerin İzolasyonu, Moleküler Karakterizasyonu ve Biyoteknolojik Potansiyelinin Belirlenmesi, Ondokuz Mayis University, Master Thesis, Samsun, Türkiye

[CR54] Kashif A, Tran LH, Jang SH, Lee C (2017) Roles of active-site aromatic residues in cold adaptation of* Sphingomonas glacialis* esterase EstSP1. ACS omega 2(12):8760–8769. 10.1021/acsomega.7b01435 31457406 10.1021/acsomega.7b01435 PMC6645578

[CR55] Kato H, Ambai S, Ikeda F, Abe K, Nakamura S, Yatsunami R (2025) Characterization of a family IV esterase from extremely halophilic archaeon* Haloarcula japonica*. Extremophiles 29(1):1–9. 10.1007/s00792-024-01370-2 10.1007/s00792-024-01370-2 PMC11614938 39625542

[CR56] Ke M, Ramesh B, Hang Y, Liu Z (2018) Engineering and characterization of a novel low temperature active and thermo stable esterase from marine *Enterobacter cloacae*. Int J Biol Macromol 118:304–310. 10.1016/j.ijbiomac.2018.05.193 29842953 10.1016/j.ijbiomac.2018.05.193

[CR57] Kelly ET, Myziuk I, Hemmings MZ, Mulla Z, Blanchet J, Ruzzini A, Berghuis AM (2026) Hydration and hydrolysis define antibiotic resistance conferred by macrolide esterases. bioRxiv 2026–2003. 10.64898/2026.03.24.713787 10.1073/pnas.2611077123 PMC13389593 42455676

[CR59] Kim YH, Cha CJ, Cerniglia CE (2002) Purification and characterization of an erythromycin esterase from an erythromycin-resistant* Pseudomonas* sp. FEMS Microbiol Lett 210(2):239–244. 10.1111/j.1574-6968.2002.tb11187.x 12044681 10.1111/j.1574-6968.2002.tb11187.x

[CR58] Kim BE, Nevitt T, Thiele DJ (2008) Mechanisms for copper acquisition, distribution and regulation. Nat Chem Biol 4(3):176–185. 10.1038/nchembio.72 18277979 10.1038/nchembio.72

[CR60] Knepp ZJ, Ghaner A, Root KT (2022) Purification and refolding protocol for cold-active recombinant esterase Aa SGNH1 from Aphanizomenon flos-aquae expressed as insoluble inclusion bodies. Prep Biochem Biotechnol 52(4):394–403. 10.1080/10826068.2021.1952601 34355672 10.1080/10826068.2021.1952601

[CR61] Kuan JE, Tsai CH, Chou CC, Wu C, Wu WF (2023) Enzymatic characterization of a novel HSL family IV esterase EstD04 from* Pseudomonas* sp. D01 in mealworm gut microbiota. Molecules 28(14):5410. 10.3390/molecules28145410 37513282 10.3390/molecules28145410 PMC10385968

[CR63] Kumar S, Stecher G, Li M, Knyaz C, Tamura K (2018) Mol Biol Evol 35(6):1547–1549. 10.1093/molbev/msy096. MEGA X: molecular evolutionary genetics analysis across computing platforms 10.1093/molbev/msy096 PMC5967553 29722887

[CR62] Kumar K, Sarkar P, Paul T, Shukla SP, Kumar S (2024) Ecotoxicological effects of triclosan on Lemna minor: bioconcentration, growth inhibition and oxidative stress. Environ Sci Pollut Res 31(45):56550–56564. 10.1007/s11356-024-34944-w 10.1007/s11356-024-34944-w 39271616

[CR64] Laemmli UK (1970) Cleavage of structural proteins during the assembly of the head of bacteriophage T4. Nature 227(5259):680–685. 10.1038/227680a0 5432063 10.1038/227680a0

[CR65] Lee YS (2016) Isolation and characterization of a novel cold-adapted esterase, MtEst45, from *Microbulbifer thermotolerans* DAU221. Front Microbiol 7:218. 10.3389/fmicb.2016.00218 26973604 10.3389/fmicb.2016.00218 PMC4773448

[CR66] Li X, Yu H, Liu S, Ma B, Wu X, Zheng X, Xu Y (2024) Discovery, characterization and mechanism of a *Microbacterium* esterase for key d-biotin chiral intermediate synthesis. Bioresources Bioprocess 11(1):59. 10.1186/s40643-024-00776-2 10.1186/s40643-024-00776-2 PMC11180644 38879848

[CR67] Li Y, Zhang Z, Xiong L, Zheng J, Zhu T, Li J, Lin A, Liu H (2025) A novel salt-adapted bifunctional glucanase/mannanase from* Klebsiella pneumoniae* and its application in oligosaccharide production. Int J Biol Macromol 296:139678. 10.1016/j.ijbiomac.2025.139678 39793794 10.1016/j.ijbiomac.2025.139678

[CR69] Liu X, Zhou M, Xing S, Wu T, He H, Bielicki JK, Chen J (2021) Identification and biochemical characterization of a novel hormone-sensitive lipase family esterase Est19 from the Antarctic bacterium* Pseudomonas* sp. E2-15. Biomolecules 11(11):1552. 10.3390/biom11111552 34827549 10.3390/biom11111552 PMC8615396

[CR68] Liu X, Zhou M, Sun R, Xing S, Wu T, He H, Chen J, Bielicki JK (2022) Characterization of a novel esterase Est33 from an Antarctic bacterium: a representative of a new esterase family. Front Microbiol 13:855658. 10.3389/fmicb.2022.855658 35655995 10.3389/fmicb.2022.855658 PMC9152352

[CR70] Liu YY, Zhang YX, Wen HM, Liu XL, Fan XJ (2023) Cloning and rational modification of a cold-adapted esterase for phthalate esters and parabens degradation. Chemosphere 325:138393. 10.1016/j.chemosphere.2023.138393 36925017 10.1016/j.chemosphere.2023.138393

[CR71] Lu M, Daniel R (2021) A novel carboxylesterase derived from a compost metagenome exhibiting high stability and activity towards high salinity. Genes 12(1):122. 10.3390/genes12010122 33478024 10.3390/genes12010122 PMC7835964

[CR72] Macomber L, Imlay JA (2009) The iron-sulfur clusters of dehydratases are primary intracellular targets of copper toxicity. Proc Natl Acad Sci 106(20):8344–8349. 10.1073/pnas.0812808106 19416816 10.1073/pnas.0812808106 PMC2688863

[CR73] Marchetti A, Orlando M, Mangiagalli M, Lotti M (2023) A cold-active esterase enhances mesophilic properties through Mn2 + binding. FEBS J 290(9):2394–2411. 10.1111/febs.16661 36266734 10.1111/febs.16661

[CR74] Martin C, Vanrolleghem PA (2014) Analysing, completing, and generating influent data for WWTP modelling: a critical review. Environ Model Softw 60:188–201. 10.1016/j.envsoft.2014.05.008

[CR75] Matrawy AA, Khalil AI, Embaby AM (2022) Molecular study on recombinant cold-adapted, detergent-and alkali stable esterase (EstRag) from *Lysinibacillus* sp.: a member of family VI. World J Microbiol Biotechnol 38(12):217. 10.1007/s11274-022-03402-5 36070019 10.1007/s11274-022-03402-5 PMC9452428

[CR76] Mazmancı B, Könen Adıgüzel S, Sadak YS, Yetkin D, Ay H, Adıgüzel AO (2023) Antimicrobial, antibiofilm, and anticancer potential of silver nanoparticles synthesized using pigment-producing *Micromonospora* sp. SH121. Prep Biochem Biotechnol 53(5):475–487. 10.1080/10826068.2022.2101001 35857430 10.1080/10826068.2022.2101001

[CR77] Méndez V, Fuentes S, Morgante V, Hernández M, González M, Moore E, Seeger M (2017) Novel hydrocarbonoclastic metal-tolerant Acinetobacter and *Pseudomonas* strains from Aconcagua river oil-polluted soil. J soil Sci plant Nutr 17(4):1074–1087. 10.4067/S0718-95162017000400017

[CR78] Milburn D, Laskowski RA, Thornton JM (1998) Sequences annotated by structure: a tool to facilitate the use of structural information in sequence analysis. Prot Eng 11:855–859. 10.1093/protein/11.10.855 10.1093/protein/11.10.855 9862203

[CR79] Mohan L, Anand S, Mittal M, Goyal K, Akanksha, Dixit A, Kumar R, Jain R, g., Diwan P (2025) Cross-sectional study: Knowledge assessment of youth regarding the global public health threat of antibiotic resistance. J Public Health 33(9):1929–1938. 10.1007/s10389-023-02179-7

[CR80] Mohanan N, Wong CH, Budisa N, Levin DB (2022) Characterization of polymer degrading lipases, LIP1 and LIP2 from *Pseudomonas chlororaphis* PA23. Front Bioeng Biotechnol 10:854298. 10.3389/fbioe.2022.854298 35519608 10.3389/fbioe.2022.854298 PMC9065602

[CR81] Mussakhmetov A, Silayev D (2025) Esterases: mechanisms of action, biological functions, and application prospects. Appl Microbiol 5(4):139. 10.3390/applmicrobiol5040139

[CR82] Nawaz MZ, Khalid HR, Mirza MU, Xu L, Haider SZ, Al-Ghanim KA, Barceló D, Zhu D (2024) Elucidating the bioremediation potential of laccase and peroxidase enzymes from *Bacillus ligniniphilus* L1 in antibiotic degradation: a computationally guided study. Bioresour Technol 413:131520. 10.1016/j.biortech.2024.131520 39321942 10.1016/j.biortech.2024.131520

[CR86] Noby N, Saeed H, Embaby AM, Pavlidis IV, Hussein A (2018) Cloning, expression and characterization of cold active esterase (EstN7) from Bacillus cohnii strain N1: a novel member of family IV. Int J Biol Macromol 120:1247–1255. 10.1016/j.ijbiomac.2018.07.169 30063933 10.1016/j.ijbiomac.2018.07.169

[CR84] Noby N, Hussein A, Saeed H, Embaby AM (2020) Recombinant cold-adapted halotolerant, organic solvent-stable esterase (estHIJ) from Bacillus halodurans. Anal Biochem 591:113554. 10.1016/j.ab.2019.113554 31863727 10.1016/j.ab.2019.113554

[CR83] Noby N, Auhim HS, Winter S, Worthy HL, Embaby AM, Saeed H, Hussein A, Pudney CR, Rizkallah PJ, Wells SA, Jones DD (2021) Structure and in silico simulations of a cold-active esterase reveals its prime cold-adaptation mechanism. Open Biol 11(12):210182. 10.1098/rsob.210182 34847772 10.1098/rsob.210182 PMC8633780

[CR85] Noby N, Johnson RL, Tyzack JD, Embaby AM, Saeed H, Hussein A, Khattab SN, Rizkallah PJ, Jones DD (2022) Structure-guided engineering of a family IV cold-adapted esterase expands its substrate range. Int J Mol Sci 23(9):4703. 10.3390/ijms23094703 35563094 10.3390/ijms23094703 PMC9100969

[CR87] OECD (2006) Test No. 221:* Lemna* sp. growth inhibition test, OECD guidelines for the testing of chemicals, Sect. 2. OECD Publishing, Paris. 10.1787/9789264016194-en

[CR88] Oharisi OOL, Ncube S, Nyoni H, Madikizela ML, Olowoyo OJ, Maseko BR (2023) Occurrence and prevalence of antibiotics in wastewater treatment plants and effluent receiving rivers in South Africa using UHPLC-MS determination. J Environ Manage 345:118621. 10.1016/j.jenvman.2023.118621 37480667 10.1016/j.jenvman.2023.118621

[CR89] Ortega-de la Rosa ND, Romero-Borbón E, Rodríguez JA, Camacho-Ruiz A, Córdova J (2024) Cloning, Expression, characterization and immobilization of a recombinant carboxylesterase from the halophilic archaeon, *Halobacterium salinarum* NCR-1. Biomolecules 14(5):534. 10.3390/biom14050534 38785941 10.3390/biom14050534 PMC11118615

[CR90] Park JE, Jeong GS, Lee HW, Kim SK, Kim J, Kim H (2021) Characterization of a novel family IV esterase containing a predicted CzcO domain and a family V esterase with broad substrate specificity from an oil-polluted mud flat metagenomic library. Appl Sci 11(13):5905. 10.3390/app11135905

[CR91] Patra M, Gupta AK, Kumar D, Kumar B (2025) Antimicrobial resistance: a rising global threat to public health. Infect Drug Resist 5419–5437. 10.2147/IDR.S530557 10.2147/IDR.S530557 PMC12558087 41158783

[CR92] Paysan-Lafosse T, Andreeva A, Blum M, Chuguransky SR, Grego T, Pinto BL, Salazar GA, Bileschi ML, Llinares-López F, Meng-Papaxanthos L, Colwell LJ, Grishin NV, Schaeffer RD, Clementel D, Tosatto SCE, Sonnhammer E, Wood V, Bateman A (2025) The Pfam protein families database: embracing AI/ML. Nucleic Acids Res 53(D1):D523–D534. 10.1093/nar/gkae997 39540428 10.1093/nar/gkae997 PMC11701544

[CR93] Pettersen EF, Goddard TD, Huang CC, Couch GS, Greenblatt DM, Meng EC, Ferrin TE (2004) UCSF Chimera-a visualization system for exploratory research and analysis. J Comput Chem 25(13):1605–1612. 10.1002/jcc.20084 15264254 10.1002/jcc.20084

[CR94] Radić S, Stipaničev D, Cvjetko P, Mikelić IL, Rajčić MM, Širac S, Pevalek-Kozlina B, Pavlica M (2010) Ecotoxicological assessment of industrial effluent using duckweed (*Lemna minor* L.) as a test organism. Ecotoxicology 19(1):216–222. 10.1007/s10646-009-0408-0 19757030 10.1007/s10646-009-0408-0

[CR95] Radić S, Stipaničev D, Cvjetko P, Rajčić MM, Širac S, Pevalek-Kozlina B, Pavlica M (2011) Duckweed Lemna minor as a tool for testing toxicity and genotoxicity of surface waters. Ecotoxicol Environ Saf 74(2):182–187. 10.1016/j.ecoenv.2010.06.011 20638723 10.1016/j.ecoenv.2010.06.011

[CR96] Rafeeq H, Hussain A, Shabbir S, Ali S, Bilal M, Sher F, Iqbal HM (2022) Esterases as emerging biocatalysts: mechanistic insights, genomic and metagenomic, immobilization, and biotechnological applications. Biotechnol Appl Chem 69(5):2176–2194. 10.1002/bab.2277 10.1002/bab.2277 34699092

[CR97] Rahman MA, Culsum U, Tang W, Zhang SW, Wu G, Liu Z (2016) Characterization of a novel cold active and salt tolerant esterase from* Zunongwangia profunda*. Enzym Microb Technol 85:1–11. 10.1016/j.enzmictec.2015.12.013 10.1016/j.enzmictec.2015.12.013 26920474

[CR98] Ren J, Ni S, Shen Y, Niu D, Sun R, Wang C, Deng L, Zhang Q, Tang Y, Jiang X, Li Z, Li C (2022) Characterization of the erythromycin degradation pathway and related enzyme in *Rhodococcus gordoniae* rjjtx-2. J Clean Prod 379:134758. 10.1016/j.jclepro.2022.134758

[CR99] Saini K, Prajapati A, Kumar SS, Kumar V, Bajar S (2026) A Review on the fate of emerging contaminants in landfill leachate: insights from conventional treatment approaches. Water Air Soil Pollut 237(5):273. 10.1007/s11270-025-08937-5

[CR100] Santos JC, Handa S, Fernandes LG, Bleicher L, Gandin CA, de Oliveira-Neto M, Ghosh P, Nascimento ALT (2023) Structural and biochemical characterization of *Leptospira interrogans* Lsa45 reveals a penicillin-binding protein with esterase activity. Process Biochem 125:141–153. 10.1016/j.procbio.2022.12.010 36643388 10.1016/j.procbio.2022.12.010 PMC9836055

[CR101] Sarkar J, Dutta A, Pal Chowdhury P, Chakraborty J, Dutta TK (2020) Characterization of a novel family VIII esterase EstM2 from soil metagenome capable of hydrolyzing estrogenic phthalates. Microb Cell Fact 19(1):77. 10.1186/s12934-020-01336-x 32209105 10.1186/s12934-020-01336-x PMC7092541

[CR102] Sha L, He WS, Zheng T, Fei Y, Fang Y, Yang H, Chen G (2024) Structure-directed bioengineering the lid1 of cold-adapted* Pseudomonas* sp. TB11 esterase to boost catalytic capacity. Int J Biol Macromol 255:128302. 10.1016/j.ijbiomac.2023.128302 37992944 10.1016/j.ijbiomac.2023.128302

[CR103] Shakiba MH, Ali MSM, Rahman RNZRA, Salleh AB, Leow TC (2016) Cloning, expression and characterization of a novel cold-adapted GDSL family esterase from *Photobacterium* sp. strain J15. Extremophiles 20:45–55. 10.1007/s00792-015-0796-4 10.1007/s00792-015-0796-4 26475626

[CR105] Singh S, Soni M, Gupta N, Sandhu P, Tripathi D, Pratap JV, Subramanian S, Manickam N (2024) Unravelling biochemical and molecular mechanism of a carboxylesterase from* Dietzia kunjamensis* IITR165 reveal novel activities against polyethylene terephthalate. Biochem Biophys Res Commun 735:150833. 10.1016/j.bbrc.2024.150833 39423573 10.1016/j.bbrc.2024.150833

[CR104] Singh D, Joshi H, Maurya S, Shukla S, Madheshiya K, Gupta G (2025) Bioremediation of antibiotics and their metabolites from waste water. Biotechnological removal of emerging pollutants from wastewater systems. Springer, Singapore, pp 223–243. 10.1007/978-981-96-3945-8_10Nature Singapore

[CR106] Sondhi S, Kaur R, Madan J (2021) Purification and characterization of a novel white highly thermo stable laccase from a novel Bacillus sp. MSK-01 having potential to be used as anticancer agent. Int J Biol Macromol 170:232–238. 10.1016/j.ijbiomac.2020.12.082 33340630 10.1016/j.ijbiomac.2020.12.082

[CR107] Soto-Hernández A, Muriel-Millán LF, Gracia A, Sánchez-Flores A, Pardo-López L (2025) Enzymatic characterization and polyurethane biodegradation assay of two novel esterases isolated from a polluted river. PLoS ONE 20(7):e0327637. 10.1371/journal.pone.0327637 40700380 10.1371/journal.pone.0327637 PMC12286390

[CR108] Tabor S (1989) DNA ligases. Curr Protoc Mol Biol 8(1):3–14. 10.1002/0471142727.mb0314s08 10.1002/0471142727.mb0314s08 18265223

[CR109] Tao H, Zhou L, Zhou Y, Wang Y, Lv H, Wang T, Xu C, Chu Y, Wang X, Song T, Lin J (2026) Functional characterization of macrolide esterase from cyanobacteria and their potential dissemination risk. npj Antimicrobials Resist 4(1):10. 10.1038/s44259-026-00182-y 10.1038/s44259-026-00182-y PMC12910000 41699255

[CR110] Trott O, Olson AJ (2010) AutoDock Vina: improving the speed and accuracy of docking with a new scoring function, efficient optimization, and multithreading. J Comput Chem 31(2):455–461. 10.1002/jcc.21334 19499576 10.1002/jcc.21334 PMC3041641

[CR111] Truongvan N, Jang SH, Lee C (2016) Flexibility and stability trade-off in active site of cold-adapted Pseudomonas mandelii esterase EstK. Biochemistry 55(25):3542–3549. 10.1021/acs.biochem.6b00177 27259687 10.1021/acs.biochem.6b00177

[CR112] Wang H, Zhang S, Lin Z, Xiao F, Xiang B, Li A (2025a) Occurrence, removal and ecological risk assessment of antibiotics in rural domestic wastewater treatment systems in the Beijing-Tianjin-Hebei region. J Hazard Mater 139127. 10.1016/j.jhazmat.2025.139127 10.1016/j.jhazmat.2025.139127 40614416

[CR113] Wang Y, Deng C, Wang X (2025b) Characterization of a novel salt-and solvent-tolerant esterase Dhs82 from soil metagenome capable of hydrolyzing estrogenic phthalate esters. Biophys Chem 316:107348. 10.1016/j.bpc.2024.107348 39531866 10.1016/j.bpc.2024.107348

[CR114] Waterhouse AM, Procter JB, Martin DM, Clamp M, Barton GJ (2009) Jalview Version 2—a multiple sequence alignment editor and analysis workbench. Bioinformatics 25(9):1189–1191. 10.1093/bioinformatics/btp033 19151095 10.1093/bioinformatics/btp033 PMC2672624

[CR115] Waterhouse A, Bertoni M, Bienert S, Studer G, Tauriello G, Gumienny R, Heer FT, de Beer TAP, Rempfer C, Bordoli R, Lepore R, Schwede T (2018) SWISS-MODEL: homology modelling of protein structures and complexes. Nucleic Acids Res 46(W1):W296–W303. 10.1093/nar/gky427 29788355 10.1093/nar/gky427 PMC6030848

[CR116] Wicka M, Wanarska M, Krajewska E, Pawlak-Szukalska A, Kur J, Cieśliński H (2016) Cloning, expression, and biochemical characterization of a cold-active GDSL-esterase of a *Pseudomonas* sp. S9 isolated from Spitsbergen island soil. Acta Biochim Pol 63(1):117–125. 10.18388/abp.2015_1074 26824293 10.18388/abp.2015_1074

[CR117] Wu G, Zhang S, Zhang H, Zhang S, Liu Z (2013) A novel esterase from a psychrotrophic bacterium *Psychrobacter celer* 3Pb1 showed cold-adaptation and salt-tolerance. J Mol Catal B Enzymat 98:119–126. 10.1016/j.molcatb.2013.10.012

[CR118] Wu G, Zhang X, Wei L, Wu G, Kumar A, Mao T, Liu Z (2015) A cold-adapted, solvent and salt tolerant esterase from marine bacterium *Psychrobacter pacificensis*. Int J Biol Macromol 81:180–187. 10.1016/j.ijbiomac.2015.07.045 26231332 10.1016/j.ijbiomac.2015.07.045

[CR119] Xing S, Xie W, Hu G, Luo C, Zhu H, He L, Li C, Wang X, Zeng X (2025) The synthesis of cinnamyl acetate and deacetyl-7-aminocephalosporanic acid by a GDSL-type esterase and its substrate specificity analysis. Enzym Microb Technol 182:110532. 10.1016/j.enzmictec.2024.110532 10.1016/j.enzmictec.2024.110532 39471645

[CR120] Xiong L, Li Y, Yu H, Wei Y, Li H, Ji X (2023) Whole genome analysis and cold adaptation strategies of *Pseudomonas sivasensis* W-6 isolated from the Napahai plateau wetland. Sci Rep 13(1):14190. 10.1038/s41598-023-41323-x 37648730 10.1038/s41598-023-41323-x PMC10468529

[CR121] Yang R, Wu J, Zhang Y, Zhang Z (2025) A novel esterase from *Burkholderia* sp. YD106 capable of hydrolysis of methyl (R, S)-N-(2, 6-dimethylphenyl) alaninate, and its mutation for improving enantioselectivity. Appl Biochem Biotechnol. 10.1007/s12010-025-05292-3 10.1007/s12010-025-05292-3 40531301

[CR122] Yao J, Gui L, Yin S (2021) A novel esterase from a soil metagenomic library displaying a broad substrate range. AMB Express 11(1):38. 10.1186/s13568-021-01198-5 33666762 10.1186/s13568-021-01198-5 PMC7936011

[CR124] Zhang S, Wu G, Liu Z, Shao Z, Liu Z (2014) Characterization of EstB, a novel cold-active and organic solvent-tolerant esterase from marine microorganism *Alcanivorax dieselolei* B-5 (T). Extremophiles 18(2):251–259. 10.1007/s00792-013-0612-y 24318107 10.1007/s00792-013-0612-y

[CR125] Zhang W, Xu H, Wu Y, Zeng J, Guo Z, Wang L, Shen C, Qiao D, Cao Y (2018) A new cold-adapted, alkali-stable and highly salt-tolerant esterase from *Bacillus licheniformis*. Int J Biol Macromol 111:1183–1193. 10.1016/j.ijbiomac.2018.01.152 29415411 10.1016/j.ijbiomac.2018.01.152

[CR126] Zhang Y, Lu W, Wang J, Chen M, Zhang W, Lin M, Zhou Z, Liu Z (2021) Characterization of EstDR4, a novel cold-adapted insecticides-metabolizing esterase from *Deinococcus radiodurans*. Appl Sci 11(4):1864. 10.3390/app11041864

[CR127] Zhang Y, Ouyang B, Chen Y, Zhang W, Guang C, Xu W, Mu W (2023) Transformation of macrolides residues by a novel erythromycin esterase C (Ere C) and safety evaluation of transformed products on *Caenorhabditis elegans*. Process Biochem 129:159–169. 10.1016/j.procbio.2023.03.008

[CR123] Zhang J, Lin L, Wei W, Wei D (2024) Identification, characterization, and computer-aided rational design of a novel thermophilic esterase from *Geobacillus subterraneus*, and application in the synthesis of cinnamyl acetate. Appl Biochem Biotechnol 196(6):3553–3575. 10.1007/s12010-023-04697-2 37713064 10.1007/s12010-023-04697-2

[CR128] Zhou J, Wu Z, Yu D, Yang L (2020) Toxicity of the herbicide flurochloridone to the aquatic plants *Ceratophyllum demersum* and* Lemna minor*. Environ Sci Pollut Res 27(4):3923–3932. 10.1007/s11356-019-06477-0 10.1007/s11356-019-06477-0 31823263

[CR129] Zieliński M, Park J, Sleno B, Berghuis AM (2021) Structural and functional insights into esterase-mediated macrolide resistance. Nat Commun 12(1):1732. 10.1038/s41467-021-22016-3 33741980 10.1038/s41467-021-22016-3 PMC7979712

