# Supplementary material for: Heterologous expression of a cold-active small esterolytic enzyme from Pseudomonas sivasensis R11S16 and its potential for azithromycin removal
Source: 3 Biotech. 2026 Jun 1;16(6):242. doi: 10.1007/s13205-026-04884-y (PMC13230378; doi:10.1007/s13205-026-04884-y)
Supplement: Supplementary file 1 — Supplementary Material 1 [file 13205_2026_4884_MOESM1_ESM.docx]

**Heterologous Expression of a Cold-Active Small Esterolytic Enzyme from *Pseudomonas sivasensis* R11S16 and Its Potential for Azithromycin Removal**

**Supplementary information 1.** The image shows DNA ladder **(Line L)** and amplified YqiA/YcfP family alpha/beta fold hydrolase (*yfh*) gene **(Line 1)**.

The *Pseudomonas sivasensis* R11S16 genomic DNA was extracted utilizing the PureLink™ Genomic DNA Mini Kit (Invitrogen™). The target YqiA/YcfP family alpha/beta fold hydrolase gene was then amplified and purified. To verify amplification, the purified product was resolved on a 0.8% agarose gel (0.5 µg/mL EtBr) at 100 V. The gel was visualized using a Gel Doc XR System (Bio-Rad Laboratories), and the resulting 600 bp band, as determined by the DNA ladder, verified the successful amplification of the YqiA/YcfP family alpha/beta fold hydrolase gene.


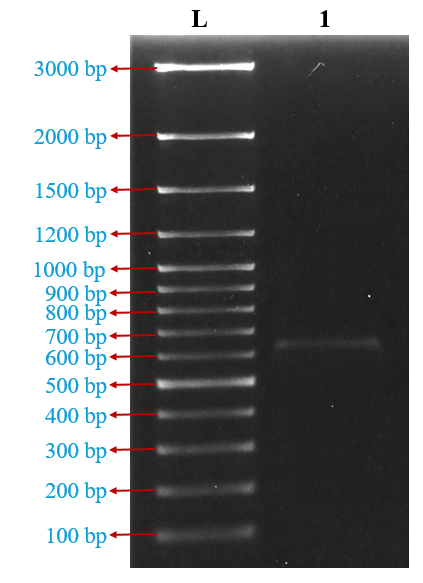


**Supplementary information 2.** The image shows DNA ladder **(Line L)**, extracted pET20b(+) **(Line 1)**, linearized pET20b(+) **(Line 2)**, digested *yfh* gene **(Line 3)**.

To construct the recombinant molecule, the plasmid pET20b(+) was extracted from E. coli DH5α cells using the FavorPrep™ Plasmid Extraction Kit (Favorgen, Pintung, Taiwan). Then, pET20b(+) and the amplified yfh gene were digested separately with the XhoI enzyme (# ER0691), according to the protocols in the user guide provided by the manufacturer. Digested DNAs were checked on the 0.8% agarose gel to verify the successful digestion.


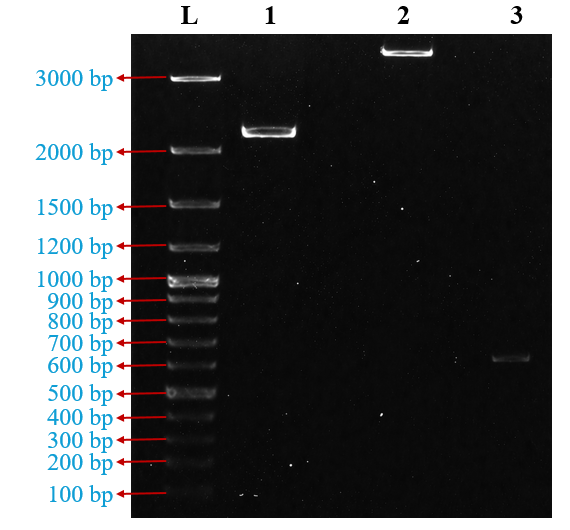


**Supplementary information 3.** Screening of clones harboring the recombinant molecule. DNA band in the red cycle indicates the recombinant molecule. The plasmids on the far left side of the gel were extracted from *E. coli* DN5α cells harboring pET20b(+) (negative control).

Following transformation, colonies observed on selective LB agar (supplemented with ampicillin) were randomly selected and subcultured using the replica plating technique. These clones were subsequently grown in liquid LB medium containing ampicillin for screening. To rapidly identify clones harboring the recombinant molecule, including the yfh gene (pet20b(+)-yfh), a rapid plasmid isolation protocol was performed (Xu-Dong et al., 2007). Briefly, 800 μL of the overnight cultures were harvested by centrifugation at 13,000 rpm for 5 minutes, and the resulting cells were resuspended in 50 μL of LB medium. An equal volume of phenol:chloroform:isoamyl alcohol (25:24:1, v/v) was added to the suspension, followed by 1 minute of vortexing and centrifugation at 20,803 x g (13,000 rpm) for 10 min. The supernatants were then analyzed via agarose gel electrophoresis to visualize plasmid bands. Clones from which the plasmid was obtained, which is positioned above the insert-free plasmid (negative control), were prioritized for further experiments.


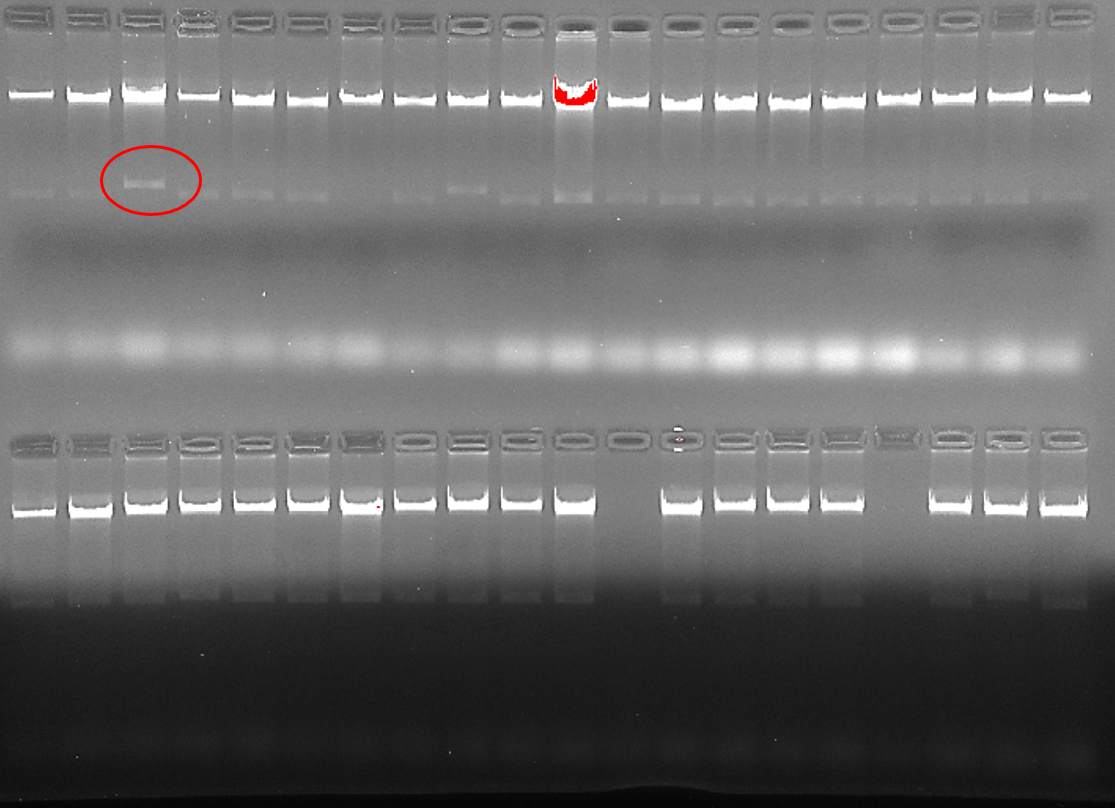


**Supplementary information 4.** Comparison between the DNA sequence of the *yfh* gene in isolate R11516 and its homolog in the type strain of *P. sivasensis*. The *yfh* gene sequence of the type strain was retrieved from NCBI (Accession No. CP176442.1). Sequence alignment was performed using Clustal Omega (<https://www.ebi.ac.uk/jdispatcher/msa>) and visualized with Jalview (Waterhouse et al., 2009). Comparative analysis revealed nucleotide differences at positions 108, 254, 256, 257, 275, and 297 in the *yfh* gene of *P. sivasensis* R11S16 relative to the type strain. Among these variations, the substitution at position 254 resulted in an amino acid change from glycine to alanine; substitutions at positions 256 and 257 caused a valine-to-glutamine replacement; and the substitution at position 275 led to the replacement of valine with glutamic acid.


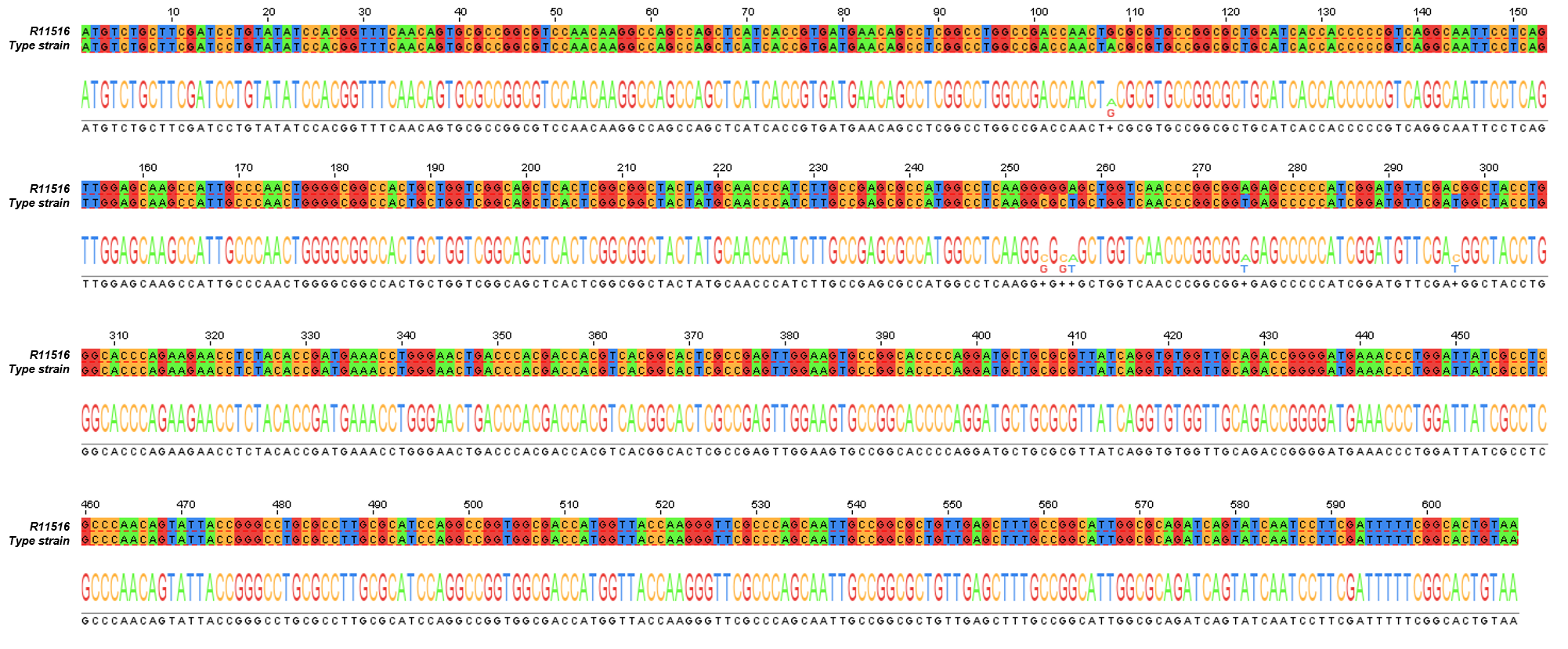


References

Xu-Dong, G. U. O., Shu-Yan, M. A. O., Dong-Xia, H. O. U., & Shorgan, B. O. U. (2007). A rapid method for preparation of plasmid DNA for screening recombinant clones. Chinese Journal of Biotechnology, 23(1), 176-178. <https://doi.org/10.1016/S1872-2075(07)60015-6>

Waterhouse, A. M., Procter, J. B., Martin, D. M., Clamp, M., & Barton, G. J. (2009). Jalview Version 2—a multiple sequence alignment editor and analysis workbench. Bioinformatics, 25(9), 1189-1191. <https://doi.org/10.1093/bioinformatics/btp033>
